# Metabolism as an early predictor of DPSCs aging

**DOI:** 10.1038/s41598-018-37489-4

**Published:** 2019-02-18

**Authors:** Dannie Macrin, Ammar Alghadeer, Yan Ting Zhao, Jason W. Miklas, Abdiasis M. Hussein, Damien Detraux, Aaron M. Robitaille, Anup Madan, Randall T. Moon, Yuliang Wang, Arikketh Devi, Julie Mathieu, Hannele Ruohola-Baker

**Affiliations:** 1 Department of Biochemistry, University of Washington, School of Medicine, Seattle, WA 98195 USA; 2 Institute for Stem Cell and Regenerative Medicine, University of Washington, School of Medicine, Seattle, WA 98109 USA; 3 Present Address: Department of Genetic Engineering, SRM Institute of Science and Technology, Chennai, 603203 India; 4 Department of Oral Health Sciences, University of Washington, School of Dentistry, Seattle, WA 98109 USA; 5 Department of Biomedical Dental Sciences, Imam Abdulrahman bin Faisal University, College of Dentistry, Dammam, 31441 Saudi Arabia; 6 Department of Bioengineering, University of Washington, Seattle, WA 98195 USA; 7 Department of Pharmacology, University of Washington, Seattle, WA 98109 USA; 8 Covance Genomics Laboratory, Redmond, WA 98052 USA; 9 Paul G. Allen School of Computer Science and Engineering, University of Washington, Seattle, WA 98195 USA; 10 Department of Comparative Medicine, University of Washington, School of Medicine, Seattle, WA 98195 USA

## Abstract

Tissue resident adult stem cells are known to participate in tissue regeneration and repair that follows cell turnover, or injury. It has been well established that aging impedes the regeneration capabilities at the cellular level, but it is not clear if the different onset of stem cell aging between individuals can be predicted or prevented at an earlier stage. Here we studied the dental pulp stem cells (DPSCs), a population of adult stem cells that is known to participate in the repair of an injured tooth, and its properties can be affected by aging. The dental pulp from third molars of a diverse patient group were surgically extracted, generating cells that had a high percentage of mesenchymal stem cell markers CD29, CD44, CD146 and Stro1 and had the ability to differentiate into osteo/odontogenic and adipogenic lineages. Through RNA seq and qPCR analysis we identified homeobox protein, Barx1, as a marker for DPSCs. Furthermore, using high throughput transcriptomic and proteomic analysis we identified markers for DPSC populations with accelerated replicative senescence. In particular, we show that the transforming growth factor-beta (TGF-β) pathway and the cytoskeletal proteins are upregulated in rapid aging DPSCs, indicating a loss of stem cell characteristics and spontaneous initiation of terminal differentiation. Importantly, using metabolic flux analysis, we identified a metabolic signature for the rapid aging DPSCs, prior to manifestation of senescence phenotypes. This metabolic signature therefore can be used to predict the onset of replicative senescence. Hence, the present study identifies Barx1 as a DPSCs marker and dissects the first predictive metabolic signature for DPSCs aging.

## Introduction

In the adult human body, stem cells are present in most of the organs in varying proportions performing the biological function of ensuring normal regeneration needed for the maintenance of the organ^1–5^. Understanding the basic molecular mechanisms that govern the regenerative capacity of adult stem cells may allow us to utilize these cells for future therapeutic approaches such as regenerative medicine and tissue engineering.

Mammalian teeth are formed during development by the interactions between the cranial neural crest derived mesoderm and the stomodeal ectoderm^6–8^. Previous studies have revealed a stem cell population that remains regenerative in adult teeth, the perivascular dental pulp stem cells (DPSC) in postnatal human dental pulp^9^. DPSCs in humans are known to be involved in regeneration of dentin structure produced by odontoblast cells^8,10–13^.

Stem cells isolated from dental pulp have been successfully differentiated into adipogenic, chondrogenic, osteogenic and odontogenic lineages^14–16^. DPSCs are thought to express mesenchymal cell surface markers such as CD44, CD45, CD73, CD90, CD146, CD29 and Stro-1^15,17–19^ and some reports suggest that they might express pluripotent markers OCT3/4, NANOG and SOX2^20^. While many studies use MSC markers to characterize these unique stem cells and attribute their differentiation capacity to the combinatorial expression of these molecular markers, no specific markers have been identified for DPSCs.

As observed with many adult stem cells, mesenchymal stem cells (MSC) from various tissues also show age-dependent decline in their regenerative capacity. Proliferation and differentiation capacities of MSCs isolated from older individuals’ bone marrow^21^, adipose tissue^22^, or teeth^23^ are significantly reduced compared to young individuals. The clinical data correlate with this notion as well. In the dental field, pulp capping is a treatment utilized by many dentists by introducing protective materials such as calcium hydroxide on an exposed vital pulp to induce the pulp cells to differentiate and produce a protective dentin-like layer on top. The success rate of this treatment after 1–5 years follow-up is reported to be significantly lower in older age groups^24–26^. This correlates with the reduced properties of DPSCs isolated from senior individuals. However, it is not clear if the different onset of stem cell aging between individuals can be predicted or prevented at an earlier stage.

While many studies have reported the common indicators of aging such as telomerase shortening, reduction in differentiation potential and cells’ morphological abnormalities, little is known about the aging mechanism and metabolic signature. We now analysed the metabolic signature in DPSCs derived from multiple individuals to characterize reliable DPSC specific signature. We showed that DPSC cell surface markers CD29, CD44, CD146 and Stro-1 are differentially expressed across individuals. We also employed assays to quantitatively measure the differentiation capabilities of these cells into osteo/odontogenic and adipogenic lineages. Through genome wide RNA seq analysis we identified homeobox protein, Barx1, as a marker for DPSCs. Using high resolution proteomic analysis, we identified markers for rapid aging DPSC populations. In particular, we showed that the TGF-β pathway and the proteins associated with regulation of cytoskeleton are upregulated in rapid aging DPSCs, indicating a loss of stem cell character and early initiation of terminal differentiation. Importantly, using metabolic flux analysis we identified how the metabolic signature differs between normal vs. rapid aging DPSCs. This metabolic signature is predictive since the differences can be observed prior to replicative senescence phenotypes.

## Materials and Methods

### Extraction and primary culture of DPSCs

Dental pulp stem cells were isolated from human third molars or deciduous teeth from 300 patients ranging from 9 to 58 years old. Teeth were collected from the University of Washington dental clinic after obtaining an informed consent from the patient or legal guardian. This study was approved by the Human Subjects Division of the University of Washington (HSD#51634-EJ) and all methods were performed in accordance with the relevant guidelines and regulations. The teeth were stored in DMEM Glutamax media (Gibco) with 10% Fetal Bovine serum (FBS) (VWR) solution immediately after tooth extraction. Each individual tooth was cut using a dremel at the cemento-enamel junction which exposed the pulp chamber. The pulp was then removed and minced into small pieces. The isolation of pulp tissue was done as previously^27^ with the slight modifications and optimization. The pulp tissue underwent an enzymatic digestion with a solution of collagenase type I (92 μM) (Sigma-Aldrich, 9001-12-1) and dispase (444 μM) (Gibco, 17105-041) for an hour at 37 °C with occasional vortexing. Digested pulp pieces were centrifuged to remove the enzyme solution and seeded into proliferation media containing DMEM Glutamax media (Gibco), 10% FBS (Invitrogen), ascorbic acid (0.1 mM) and 1X antibiotic antimycotic solution (Sigma-Aldrich) in a 35-mm cell culture plate. The DPCS292 was isolated from an intact deciduous tooth that was surgically extracted from a nine-year-old patient by the dentist. Pulp tissues from the deciduous teeth were isolated with the same protocol and seeded in proliferation media supplemented with insulin growth factor (IGF-1) to increase proliferation success. Once seeded, the pulp pieces were incubated at 37 °C with 5% CO_2_ with media changed every 3 days until cells migrated out and optimal confluence was achieved. When the cells were ~70% confluent, they were trypsinized and passaged to allow expansion in a 10 cm plate for cryopreservation or further experimentation. The passaged DPSCs were cultured and maintained in media containing 10% FBS and 1% Penicillin-Streptomycin (Invitrogen) in DMEM. The cell lines that were selected for further studies DPSC 29, DPSC 43 and DPSC 44 orginate from female donors, while DPSC 45 orginate from a male donor.

### Cell Culture

HeLa (ATCC), human foreskin fibroblasts (HFFs)^28^, DPSCs (PT-5025) and MSCs derived from bone marrow (MSC, PT-2501) cell lines (Lonza) were cultured with the same media composition as DPSCs later passages (10% FBS and 1% Penicillin-Streptomycin in DMEM). Human embryonic stem cells (hESCs) [Elf-1(NIH_hESCs Registry #0156)] were cultured as previously described in hESCs media^29^. Briefly, cells were grown in either mTeSR1 media (StemCell Technologies) or 2iL-I-F media (DMEM/F-12 media supplemented with 20% knock-out serum replacer (KSR), 0.1 mM nonessential amino acids (NEAA), 1 mM sodium pyruvate, penicillin/streptomycin (all from Invitrogen, Carlsbad, CA), 0.1 mM β-mercaptoethanol (Sigma-Aldrich, St. Louis, MO), 1 µM GSK3 inhibitor (CHIR99021, Selleckchem), 1 µM of MEK inhibitor (PD0325901, Selleckchem), 10 ng/mL human LIF (Chemicon), 5 ng/mL IGF1 (Peprotech) and 10 ng/mL bFGF. In one differentiation experiment, the DPSCs were primed for short term (4 days) with TeSR media supplemented with a metabolite-cocktail (TeSRmeta) containing kynurenine (KY) and methylnicotinamide (MNA), which was previously shown to be beneficial to stem cells^29^.

### Protein isolation and Western blotting

Protein extraction and Western blot analysis were performed following previously described procedures^30^. Proteins were isolated from 80% confluent plate by direct lysis with a lysis buffer containing 20 mM Tris-HCl (pH 7.5), 150 mM NaCl, 15% Glycerol, 1% Triton, 3% SDS, 25 mM β-glycerophosphate, 50 mM NaF, 10 mM Sodium Pyrophosphate, 0.5% Orthovanadate, 1% PMSF (all chemicals were from Sigma-Aldrich, St. Louis, MO), 25 U Benzonase Nuclease (EMD Chemicals, Gibbstown, NJ) and protease inhibitor cocktail (Pierce™ Protease Inhibitor Mini Tablets, Thermo Scientific, USA). Total protein was quantified by Bradford’s assay and 10 μg of protein extracts were loaded, separated by either 7.5% SDS-PAGE, or by 4–20% Mini-PROTEAN TGX gels (Bio-Rad Laboratories, Hercules, California, United States. Cat. #456-1094) and transferred to nitrocellulose membrane (0.2 μM, Bio-Rad Laboratories, Hercules, California, United States). Membranes were blocked with 5% non-fat dry milk for at least 60 minutes at room temperature and incubated overnight at 4 °C with primary antibody. The next day, the membranes were incubated for one hour with horseradish peroxidase-conjugated secondary antibodies, and then visualized using Immobilon Western Chemiluminescent HRP Substrate (Millipore Corp, Billerica, MA). Primary antibodies used were CD29 (Abcam 1:1000), CD146 (Abcam, Cambridge, MA; 1:1000), BARX1 (Santa Cruz Biotechnology, sc-81956, 1:200) and ßIII tubulin (Promega, 1:1000).

### Flow Cytometry

Cells grown to an optimal confluence of 70% were used for FACS analysis. Single cell suspension was prepared by dissociating the cells using 0.05% Trypsin and triturating in PFN (PBS + 5%FBS + 0.1%Sodium Azide). The cells were fixed with 4% paraformaldehyde in PFN over ice for 1 hour. The fixed cells were incubated with two primary antibodies per experiment (CD29[Abcam, 1:100]/CD146[Abcam, 1:50 and CD44[Abcam, 1:50]/Stro1[R&D Systems, 1:50]) at 4 °C overnight. Subsequently, the cells were incubated with fluorescent secondary antibodies over ice for 1 hour and analysed by flow-cytometer (Canto II, BD Biosciences). FlowJo software was used for gating and further analysis.

### Directed differentiation

DPSCs were cultured in 6 well plates for a period of 4–7 days until they reached 70% confluency and differentiated into adipogenic and osteo/odontogenic cells using different conditions supplemented in media. A serum free media (SFM) (Supplementary Table S1) was used as the base media for differentiation. The cells were treated with differentiation media for 10 days for osteo/odontogenesis (media components^31,32^: Supplementary Table S2) and 7 days for adipogenesis (media components^32^: Supplementary Table S3) with media change every 3 days.

### Osteo/odontogenesis quantification assay

The candidate cell lines (29, 44, 43 and 45) were cultured in osteo/odontogenic differentiation media for 10-days, followed by fixation with 4% paraformaldehyde in PBS and stained with DAPI. After DAPI quantification, the cells were stained with Alizarin Red stain (binds to extracellular calcifications). The alizarin stain was released with 10% acetic acid and neutralized with 0.1 M ammonium hydroxide. The staining intensity was then quantified in Wallac EnVision system and the relative absorption was calculated by normalizing the alizarin dye absorption with the DAPI emission.

### Adipogenesis quantification assay

Cells were incubated in the adipogenic medium for 10 days. The media was removed, and the cells were fixed with 4% paraformaldehyde for 10 minutes. The excess paraformaldehyde was washed off with PBS. 1X BODIPY stain was added and incubated in the dark for 5 minutes. The excess BODIPY stain was removed and rinsed with PBS. The cells were counter stained with DAPI and visualized under fluorescent microscope (Zeiss). Alternatively, after the 7-days adipogenic differentiation, cells were fixed with 4% paraformaldehyde in PBS and stained with DAPI. The total DAPI emission was quantified in a Wallac EnVision multiplate reader. Then the cells were stained with Oil Red O (ORO) (binds to the neutral lipids). The ORO stain was released by dissolving in 100% isopropyl alcohol for 10 min on a rocker. 80% of the total extraction volume was used for colorimetric quantification with the Wallac EnVision system, and the relative absorption was calculated by normalizing the ORO absorption with the DAPI emission.

### RNA extraction and RT-qPCR analysis

RNA was extracted using Trizol (Life Technologies) according to manufacturer’s instructions. RNA samples were treated with Turbo DNase (Thermo Fisher Scientific) and quantified using Nanodrop ND-1000. Reverse transcription was performed using iScript cDNA Synthesis Kit (Bio-Rad). 10 ng of cDNA was used to perform qRT-PCR using SYBR Green (Applied Biosystems) or TaqMan (Applied Biosystems) on a 7300 real time PCR system (Applied Biosystems). The PCR conditions were set up as the following: stage 1 as 50 °C for 2 mins, stage 2 as 95 °C for 10mis, 95 °C for 15 sec, 60 °C for 1 min (40 Cycles). ß-actin was used as an endogenous control. The primer sequences used in this work are shown in Supplementary Table S4.

### Transcriptomic data analysis

RNA was isolated from freshly revived cell samples (Passage 3–5) of the candidate cell lines and forwarded/directed for transcriptome analysis to Covance Genomics lab. RNA-seq were aligned to Ensembl GRCh37 using Tophat (^33^, version 2.0.13). Raw RNA-seq reads from this study and others^29,34–42^ were used. Gene-level read counts were quantified using FeatureCounts^43^ using Ensembl GRCh 37 gene annotations. *Prcomp* function from R was used to for Principal Component Analysis. DESeq^44^ was used for differential gene expression analysis. TopGO R package^45^ was used for Gene Ontology enrichment analysis, as well as DAVID 6.8 online analytic tool^46,47^. SPIA R package^48^ was used for signalling pathway impact analysis utilizing pathway topology data downloaded from KEGG’s website^49^ on: 3/25/2018. RNA-seq datasets generated for this study are available in the NCBI GEO database under accession number (GSE123973).

### Overexpression of BARX1 and validation of BARX1 antibody in HeLa cells

HeLa cells were transfected overnight with 2 μg/ml of a pcDNA3.1^(+)^ plasmid, constitutively expressing BARX1 under the control of CMV promoter, supplied by Genescript (CloneID: OHu15603). The transfection was carried out with Lipofectamine 3000 transfection kit (Thermo Fisher Scientific). Protein isolation and quantification was done after 48 h as described in protein isolation and western blotting section. DPSC Lonza were transfected with electroporation (Amaxa Nucleofector device) with 2 μg/ml of the same plasmid. Transfected HeLa cells and DPSC Lonza were plated in a 4-well glass chamber slide system (Thermo Fisher Scientific) overnight and fixed the following day as described in immunostaining and confocal imaging section.

### Immunostaining and confocal imaging

Cells were fixed in 4% paraformaldehyde in PBS for 15 minutes, and blocked in 2% BSA, 0.1% Triton X-100 in PBS. The cells were then incubated in Anti-BARX1 (1:100, Santa Cruz Biotechnology, sc-81956) overnight. The cells were washed with PBS and incubated with Alexa 488-conjugated secondary antibody (Molecular Probes, 1:500) in 1 hr. DAPI (Nuclear stain) and Alexa Flour 568 phalloidin (Selectively stains F-Actin, 1:250, Thermo Fisher Scientific) were used as counter stains and mounted with 2% of n-Propyl Gallate in 90% Glycerol + PBS solution. Analysis was done on a Leica TCS-SPE Confocal microscope using a 40x objective and Leica Software.

### SeaHorse Extracellular Flux assay

DPSCs, MSCs and hESCs were seeded on a 96 well seahorse plate at 2 × 10^4^ or 4 × 10^4^ cells/well. The following day culture medium was replaced with base medium (Agilent Seahorse XF base medium, USA) and 1 mM Sodium pyruvate (Gibco). Then, based on the assay type, the media was supplemented with 25 mM glucose (mitostress assay), 25 mM glucose and 50 μM carnitine (palmitate assay) or 2 mM glutamine (glucose stress assay) 1 hour before starting the assay. The cells were treated with various substrates and selective inhibitors. Mitostress: oligomycin (2.5 μM), FCCP (1 μM), rotenone (2.5 μM) and antimycin (2.5 μM). Palmitate assay: palmitate (50 μM in BSA), BSA and ETO (50 μM). Glucose assay: glucose (2.5 mM), oligomycin (1 μM) and 2-DG (50 mM). The OCR values were then normalized with readings from Hoechst staining which corresponded to the number of cells in the well.

### Cell surface area measurements

Bright-field images of cell culture were taken with ZEISS Axio Observer inverted microscope equipped with AxioCam MR R3 camera sensor. Images were processed by manually drawing a line around the cells and then individual cell area were measured with Fiji software distribution of ImageJ v1.51n^50,51^.

### Senescence assay

Analysis of cellular senescence was carried out using a Cellular Senescence Live Cell Analysis Assay Kit (Enzo Life Sciences, NY, USA. Catalog number: ENZ-KIT130-0010). Briefly, cells were treated with pretreatment solution at 37 °C for 2 h. Next, senescence-associated β-galactosidase (SA-β-gal) substrate solution was added to the cells overnight. The stained cells were trypsinized and washed twice in PBS containing 2% FBS. Cells were analysed by flow-cytometer (Canto I, BD Biosciences) and FlowJo software (TreeStar, Ashland, OR, USA).

### Proteomics

Cells were analysed as described previously^52,53^ with minor modifications. Briefly, cells were lysed in 1 M urea, 50 mM ammonium bicarbonate, pH 7.8, and extracted proteins were quantified with a BCA (Bicinchoninic Acid) assay. Proteins were reduced with 2 mM DTT, alkylated with 15 mM iodoacetamide, and digested overnight with trypsin. The resulting peptides were desalted on Waters Sep-Pak C18 cartridges. Peptides were analysed by nano-LC-MS/MS on a Fusion Orbitrap (Thermo Fisher Scientific). Peptides were separated online by reverse phase chromatography using a heated 50 °C 30 cm C18 columns (75 mm ID packed with Magic C18 AQ 3 μM/100 Α beads) in a 280 min gradient (1% to 45% acetonitrile with 0.1% formic acid) separated at 250 nL/min. The Fusion was operated in data-dependent mode with the following settings: 60000 resolutions, 350–1500 m/z full scan, Top Speed 2 seconds, and a 1.8 m/z isolation window. Identification and label free quantification of peptides were done with MaxQuant 1.5.7.4 using a 1% false discovery rate (FDR) against the human Swiss-Prot/TrEMB database downloaded from Uniprot on June 2^nd^, 2016. The databases contained forward and reverse human sequences as well as common contaminants. Peptides were searched using a 5 ppm mass error and a match between run window of 2 min. The mass spectrometry proteomics data have been deposited to the ProteomeXchange Consortium via the PRIDE partner repository with the dataset identifier PXD011962.

### String analysis

Protein-protein interaction networks were generated using the STRING software version 10.5^54^. A list of proteins found in the proteomics analysis were analysed in the software to show the known and predicted interactions under medium confidence settings.

### Secretome prediction analysis

DPSCs transcriptomic data were submitted for secretome prediction analysis by using the online tool of the Vertebrate Secretome Database (VerSeDa)^55^. The VerSeDa algorithms analyse the amino acid sequences, calculate the probability of secretion by classical or non-classical mechanisms, and return a list of predicted secretome. All default settings were used in our analysis.

## Results

### Previously identified MSC markers are differentially expressed across DPSC lines generated from patients

**Figure 1:**
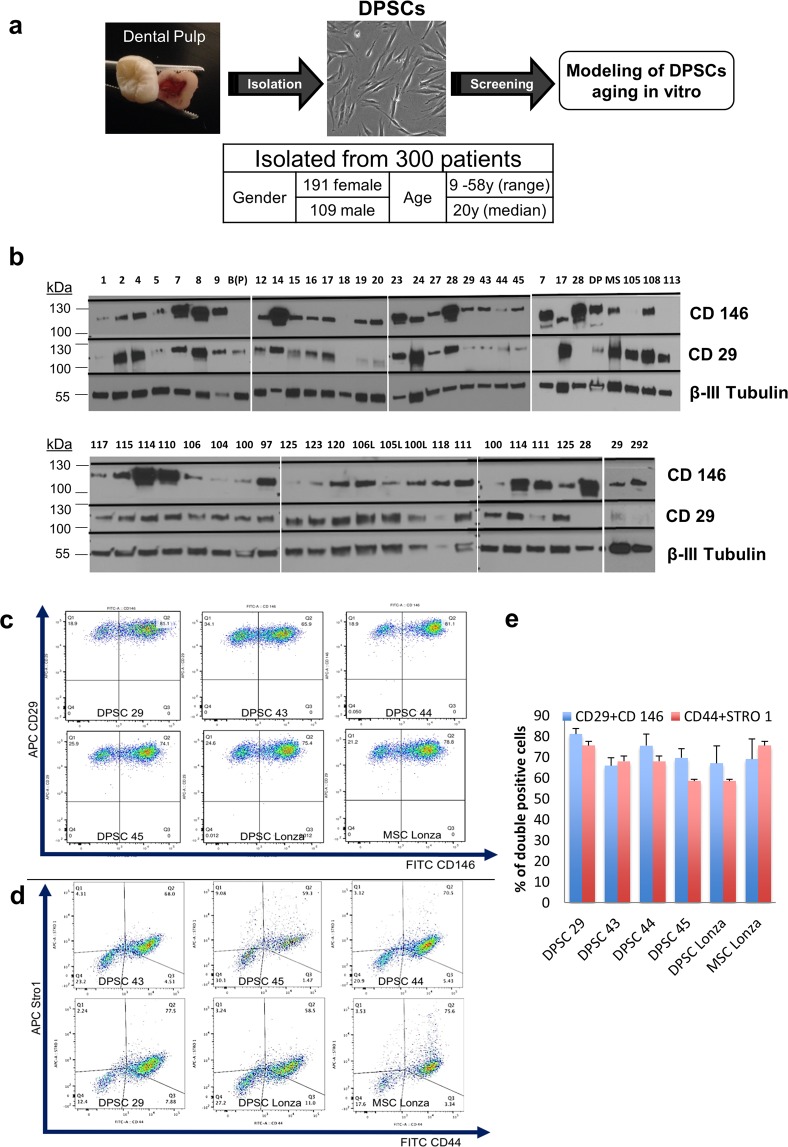
Extraction summary and preliminary screening. (**a**) A simplified summary of the isolated DPSC lines and the initial project design to model DPSCs aging *in vitro*. (**b**) Preliminary screening of cell lines with cell surface markers, CD29 and CD146 using western blot shows differential expression of these markers. (**c**,**d**) Fluorescent cytometry of candidate cell lines (DPSC 29,44,43,45) demonstrates high percentage of mesenchymal markers; CD29+CD146+ cells (**c**) and CD44+Stro1+ cells (**d**). (**e**) A representative graph shows that the percentage of “double positive” were comparable with commercial DPSC and MSC cell lines from Lonza. (n = 3 per cell line). Graph error bars are the means ± standard error of the mean (SEM).

We isolated DPSCs from dental pulp extracted from the human third molars or surgically obtained deciduous teeth of 300 patients (Fig. 1a). Some of the samples were used to optimize the protocol by identifying the window of time at which the samples could be stored in DMEM media (4 °C) prior to DPSCs isolation, without loss of DPSCs viability. The samples that took more than 20 days to reach confluence for the first passage were considered unviable. We observed that the percentage of viability and proliferation capacity of the cells decreased substantially when the pulp isolation was delayed for more than 2 days after extraction of the tooth (Supplementary Fig. S1).

The DPSCs were expanded and passaged at least twice before protein isolation or cell harvest. Western blot analysis of previously identified MSC surface markers (CD29 and CD146,^56^) were used as a means of preliminary molecular screening. We observed heterogeneity in the expression pattern of these markers (Fig. 1b). A candidate set of DPSCs (DPSC 29, 43, 44, 45), which showed mid-level expression of the CD29 and CD146 markers were selected for further analysis. We used this subgroup to test if DPSC with similar CD29 and 146 levels might show different kinetics of senescence *in vitro*. Differential potential for replicative capacity would give us tools to analyse the process in detail.

To know the actual percentage of mesenchymal cells present in the cell lines, the candidate DPSC cell lines along with commercial DPSCs and MSCs (Lonza) were analysed by flow cytometry. The results showed that our population of DPSCs had high percentage of CD29 and CD146, CD44 and Stro-1 double positive cells; 81.1%, 81.1%, 74.1% and 75.6% of CD29^+^146^+^ (Fig. 1c,e) and 77.5%, 68.5%, 59.3%, 58.5% of CD44^+^Stro-1^+^ (Fig. 1d,e) cells in DPSC 29, 44, 43 and 45, comparable to 78.8% and 75.6% of double positive cells in commercial DPSC. The results validate our isolation protocol and infer that our stem cell lines have high percentage of mesenchymal cells (Fig. 1c–e).

**Figure 2:**
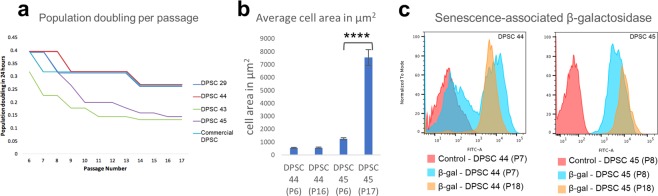
Difference in growth kinetics within the candidate cell lines. (**a**) Number of population doublings in 24 hours calculated in each consecutive passage indicates that two of the candidate cell lines demonstrated replicative senescence which is a classical hallmark of *in vitro* ageing (Cells were passaged to a density of 1:3 in each passage). (**b**) Quantification of average cell surface area in early and late passages of DPSC 44 and 45. Significance was determined by unpaired Student’s t-test; n = 70 cells per cell line were measured; ****p < 0.0001; Graph error bars are the means ± SEM (**c**) flow cytometric analysis of senescence-associated-β-gal activity in early and late passages of DPSC 44 and 45.

### Rapid and slow aging DPSC cell lines

In order to study the onset of aging in DPSCs we assessed the proliferation capacity of the cell lines through multiple passages. Interestingly we found dramatic differences between the DPSC lines extracted from different individuals. We found that DPSC 29, 44 and commercial DPSC showed minimal changes in proliferation capacity whereas DPSC 43 and 45 exhibited a dramatic decrease in proliferation with progressive cell divisions (Fig. 2a). The difference in the decrease in proliferation capacity was not caused by the donor age since the analysed cell lines (DPSC 29, 44, 43 and 45) were all isolated from individuals with similar age (21–23 years old). Based on the rate these cell lines reached replicative senescence they were classified into slow aging (SA: DPSC 29 and 44) and rapid aging cell lines (RA: DPSC 43 and 45). One of the manifestations of senesced cells is an increase in cell size^57^, therefore we measured the surface area of DPSC 45, and noted a significant (5 fold; p < 0.0001) increase in average cell size between early passage (P6) and late passage (P17), supporting DPSC 45 senescence during this time period (Fig. 2b). However, DPSC 44 showed no significant change in cell size between early passage (P6) and late passage (P16), correlating well with our classification as slow aging cell line. We further analysed the cell lines by measuring the activity of senescence-associated β-galactosidase (SA-β-gal)^58^ in a flow cytometric based assay (Fig. 2c). Two populations were observed in DPSC 44 early passage (P7) with SA-β-gal assay. At later passage (P18) in DPSC 44, most of the cells expressed SA-β-gal at high intensity. In DPSC 45, both early (P8) and late (P18) passage cells showed high intensity of SA-β-gal suggesting that senescence starts early in rapid aging cell lines.

### DPSC lines are heterogeneous in their differentiation capacities

**Figure 3:**
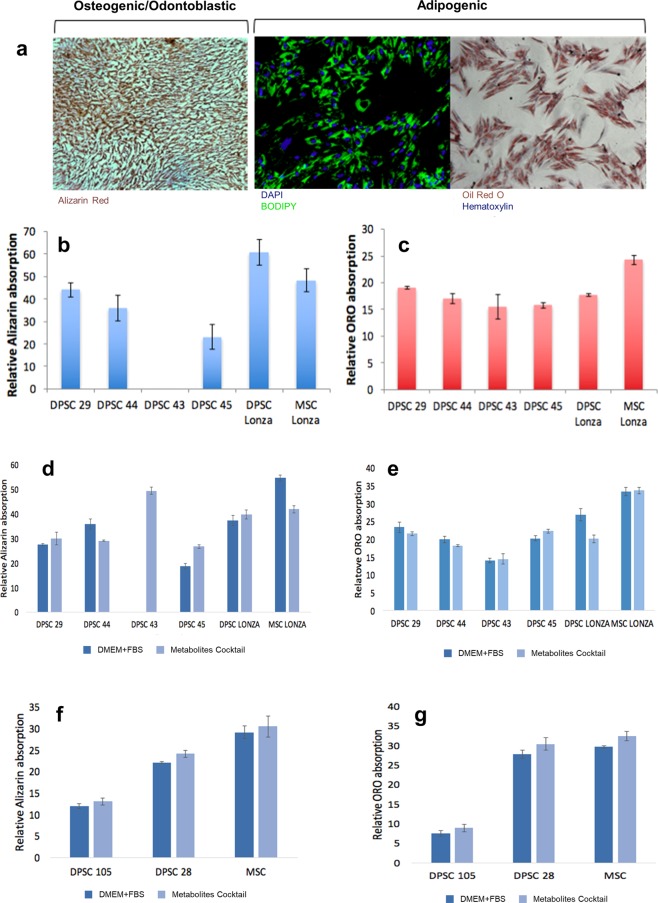
Osteo/odontogenic and Adipogenic potential of DPSCs. (**a**) Osteo/odontogenic cells were stained for extracellular calcifications with Alizarin red stain. Adipogenic cells were stained for neutral lipids with BODIPY and Oil Red O stain. (**a**) Spectrometric quantification of Alizarin stain normalized with cell numbers (DAPI staining) showing all the candidate cell lines were able to differentiate into osteoblasts/odontoblast at different levels except DPSC 43 which did not survive the osteogenic differentiation. (**c**) Spectrometric quantification of Oil Red O stain normalized with cell numbers (DAPI staining) showing all the cell lines were able to differentiate into adipocytes at different levels and are highly resembling the differentiation levels of commercial cell lines. (**d**–**g**) Treatment with TeSRmeta for 4 days did not have any impact on the differentiation potential of the candidate cell lines, except for DPSC 43 in which it enhanced the survivability and differentiation to osteoblasts/odontoblasts. (n = 3 per cell line). Graph error bars are the means ± SEM.

The multipotency of an adult stem cell can only be gauged by its ability to differentiate into multiple lineages. To analyse the multipotency of the isolated cell lines, the candidate cell lines were pushed towards adipogenic and osteo/odontogenic lineages. The cells were cultured in differentiation media for a time period of 7 days for adipogenesis and 10 days for osteogenesis/odontogenesis (Fig. 3a). The differentiation was then quantified using a cell number normalized assay or qPCR (Fig. 3b–g; Supplementary Fig. S2). Osteogenesis/odontogenesis was quantified based on the staining of extracellular calcification by alizarin red. We observed comparable osteogenic/odontoblastic differentiation between the selected candidate DPSC lines (DPSC 29, 44, and 45). However, DPSC 43 did not survive the osteogenic/odontoblastic differentiation despite many repeated trials (Fig. 3b). An analogous experiment for adipogenesis was quantified based on the staining of neutral lipid vesicles by oil red O stain. This analysis showed that all of our candidate DPSC lines (DPSC 29, 44, 43, 45) differentiated into the adipogenic lineage, similarly to the commercial control DPSC line (Fig. 3c). In an attempt to normalize the heterogeneity in differentiation, we studied the effect of a short term (4 days) treatment of the cells with a TeSR media supplemented with a metabolite-cocktail (TeSRmeta) containing kynurenine (KY) and methylnicotinamide (MNA), which was previously shown to be beneficial to stem cells^29^. While this treatment did not show a significant increase in differentiation in most of the DPSC lines, TeSRmeta treated DPSC 43 did show a dramatic increase in the osteogenic/odontoblastic differentiation (Fig. 3d–g). The molecular mechanism underlying this phenomenon is a topic of further investigation. Since many of the original lines had variation in the CD146 expression (Fig. 1b), we also differentiated two cell lines with either no CD146 expression (DPSC 105) or high level of CD146 expression (DPSC 28). We observed that the cells with the former phenotype showed minimal differentiation capacity. Though the expression levels of CD146 was not coercive to differentiation of a cell type, complete absence of CD146 led to impaired differentiation ability in both osteogenic/odontogenic and adipogenic differentiation test (Fig. 3f,g). We also examined Runx2 (osteoblast/odontoblast marker) and LPL (adipogenic marker) expression levels in differentiated cells (Supplementary Fig. S2)^59,60^. Taken together, our data showed that the analysed DPSC lines were able to differentiate into adipogenic and osteogenic/odontoblastic lineages.

### DPSCs exhibit a unique transcriptome profile

**Figure 4:**
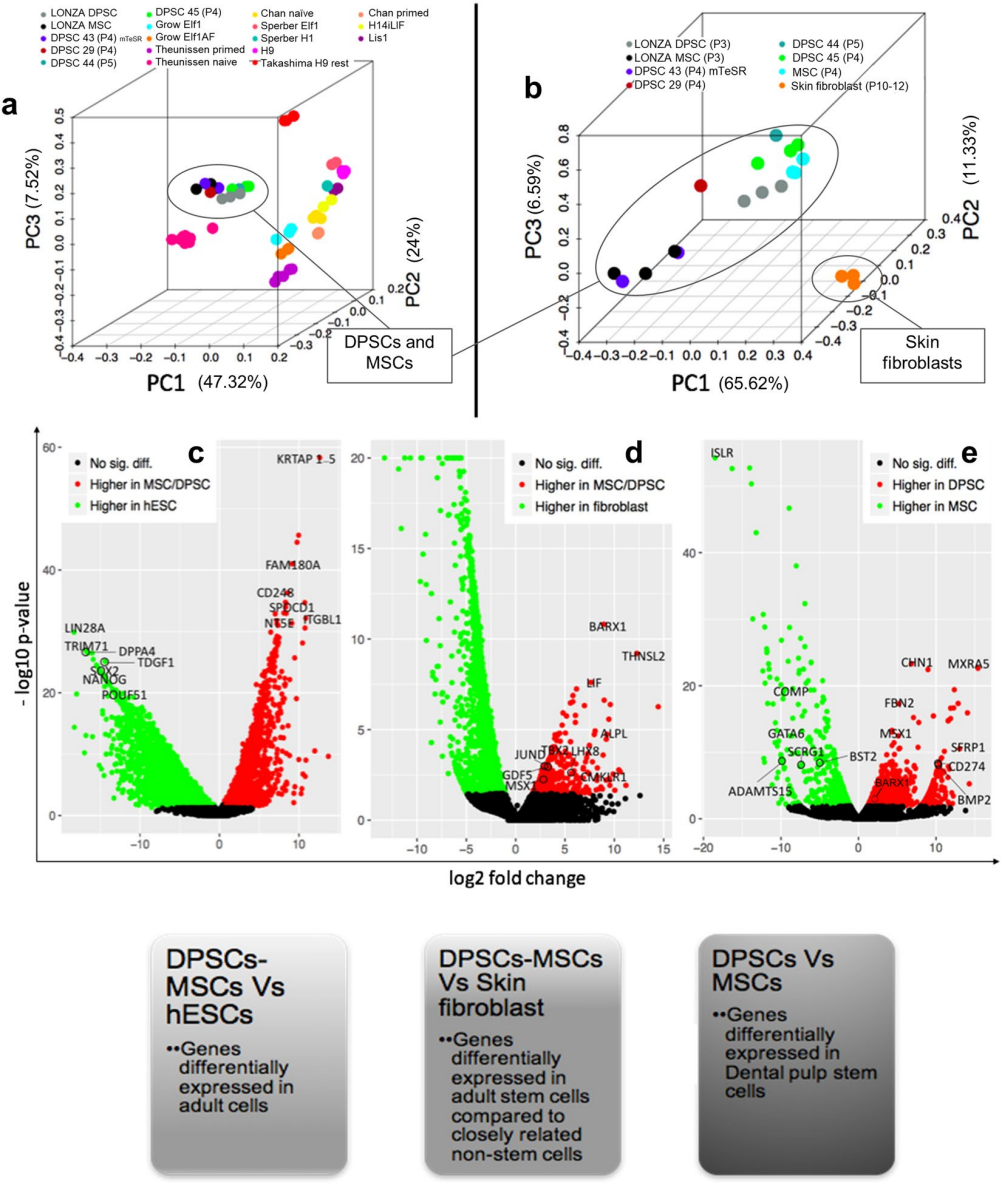
The gene expression of DPSCs compared to MSCs, Fibroblasts and hESCs. (**a**) Principal component analysis (PCA) of DPSCs, MSCs and hESCs showed that DPSCs and MSCs grouped together. (**b**)Principal component analysis (PCA) of DPSCs with skin fibroblasts and MSCs from literature showed that DPSCs have distinct expression profiles from foreskin fibroblasts. (**c**) Volcano plot of genes differentially expressed in MSCs/DPSCs vs. hESCs. (**d**) Volcano plot of genes differentially expressed in MSCs/DPSCs compared to fibroblasts. (**e**) Volcano plot of genes differentially expressed in DPSCs vs. MSCs.

Transcriptome analysis was performed to identify important genes specifically expressed in adult DPSCs. To achieve this goal, we systematically compared the gene expression signature between embryonic stem cells (hESCs) and adult stem cells derived from bone marrow and dental pulp (MSCs and DPSCs) (Fig. 4c). Similarly, the adult MSCs and DPSCs were compared with human skin fibroblasts (Fig. 4d) to identify the genes that may be responsible for the stem cell properties of these cells. Finally, the DPSCs were compared with bone marrow MSCs (Fig. 4e) to dissect the unique genes expressed in DPSCs. The genes with false discovery rate <0.1 and fold change >1.5 were considered as differentially expressed. Principal component analysis (PCA) indicated that the candidate DPSC cell lines clearly segregated from hESCs and co-localized with MSCs (Fig. 4a). PCA with skin fibroblasts, MSCs and DPSCs indicated clustering within cell types (MSCs and DPSCs separated from fibroblasts) with minimal sample to sample variability (Fig. 4b).

The comparison between hESCs and MSCs/DPSCs identified MSCs/DPSCs enriched genes, including MSC surface markers CD248 (Endosialin), CD73 (ecto-5′-nucleotidase), CD29 (ITGB1,Integrin beta 1)^61,62^, (Fig. 4c, Supplementary Table S5, Supplementary Dataset Sheet 1). We further compared our data between MSCs/DPSCs and human skin fibroblasts which revealed differentially expressed genes, such as ALPL(Alkaline Phosphatase) and GDF5 (Growth Differentiation Factor 5) which promote mineralization and osteogenic potential^63,64^, as well as negative regulators of cellular senescence TBX2 and JUND^65,66^ (Fig. 4d, Supplementary Table S6, Supplementary Dataset Sheet 2). Furthermore, the comparison between DPSCs and BM-MSCs revealed the unique genes expressed in DPSCs (Fig. 4e, Supplementary Table S7, Supplementary Dataset Sheet 3). Interestingly, both MSX1, a protein responsible for osteogenic differentiation of DPSCs^63^, and BARX1 (BARX Homeobox 1), a gene primarily known for its role in patterning of teeth^6,67–69^ were differentially up-regulated in DPSCs compared to either fibroblasts, hESCs or BM-MSCs.

### BARX1 as a DPSCs marker

The differentially-expressed gene in DPSCs compared to fibroblasts, BARX1 (Fig. 4d) is a transcription factor, part of the homeobox gene group responsible for early developmental patterning involving craniofacial development, teeth premordia and stomach specification from gut endoderm^70^ and repression of cell migration in the context of cancer^71^.

**Figure 5:**
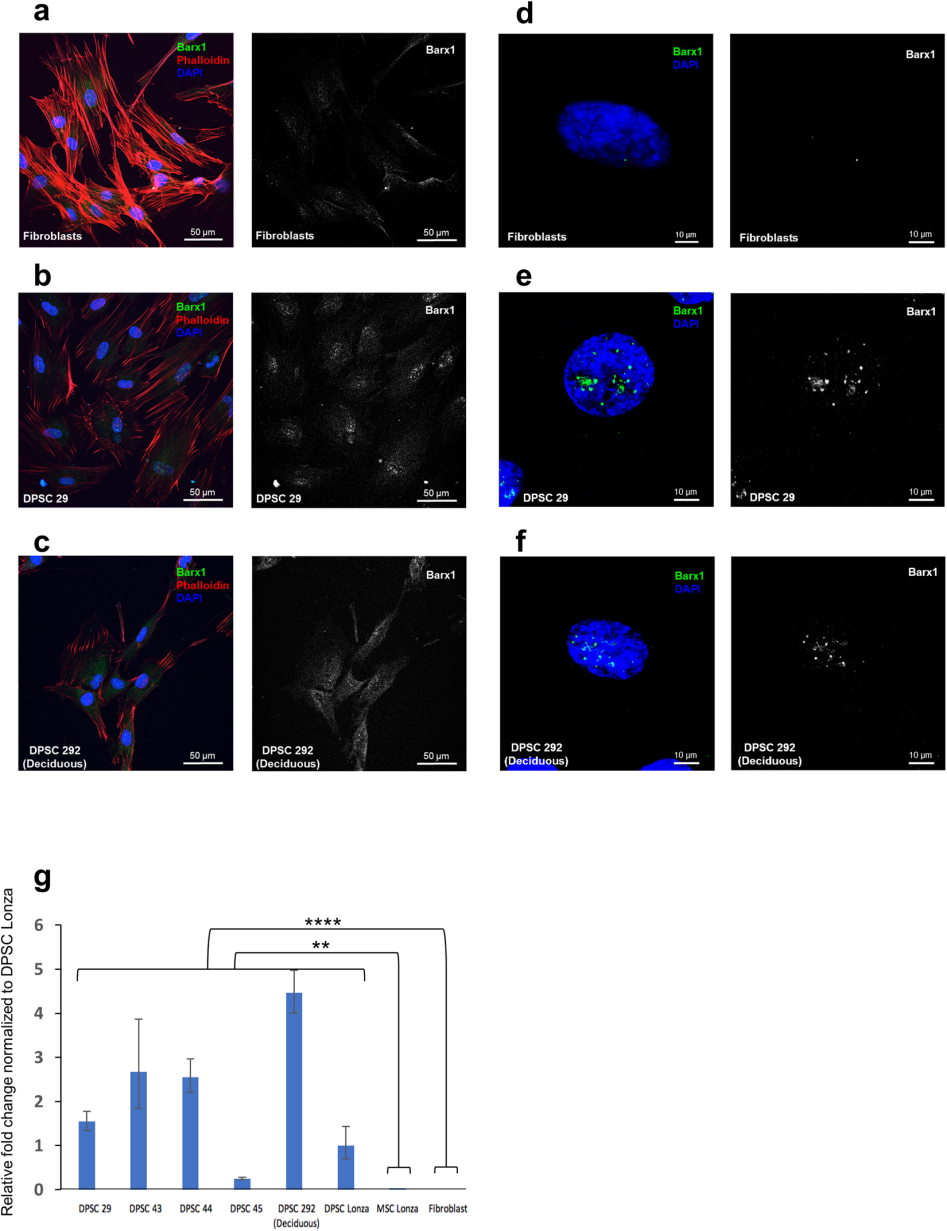
BARX1 gene as a new specific marker for DPSCs. (**a**–**c**) Immunofluorescent staining of Barx1 in skin fibroblasts, adult DPSCs (DPSC 29), and deciduous DPSCs (DPSC 292) at low power magnification. (**d**–**f**) Showing the nuclear localization of Barx1 transcription factor at high power magnification. Exposure settings and laser intensity of the confocal microscope were adjusted and normalized for fibroblasts, and same settings were used for the DPSCs. (**g**) Quantitative PCR reveals absence of BARX1 gene in BM-MSCs and in skin fibroblasts. Fold change normalized to DPSC Lonza. Significance was determined by unpaired Student’s t-test; n = 3–6 per cell line; **p < 0.01; ****p < 0.0001; Graph error bars are the means ± SEM.

To validate the RNA-seq results, we analysed BARX1 transcript and protein expression with RT-qPCR and immunofluorescence. The Barx1 antibody was validated on HeLa cells transfected with BARX1 overexpression vector (Supplementary Fig. S3). We next performed BARX1 immunofluorescence staining for DPSCs 29, DPSCs 292 and human skin fibroblasts (HFF-1) cell lines (Fig. 5). Our results showed that Barx1 was localized in the nuclei of DPSCs as a punctate pattern, suggesting that Barx1 binds to specific chromosomal locations (Fig. 5b,c,e and f). Importantly, skin fibroblasts showed no nuclear Barx1 protein signal in immunofluorescence (Fig. 5a,d) and BM-MSCs (Lonza) expressed a low level of Barx1 (Supplementary Fig. S3). The BARX1 transcript qPCR analysis supported the finding; DPSCs showed BARX1 expression while fibroblasts did not express BARX1 gene (Fig. 5g). These data suggest that BARX1 is a DPSCs marker that can discriminate against fibroblast population.

### Metabolic signature, predictive of the rapid aging

**Figure 6:**
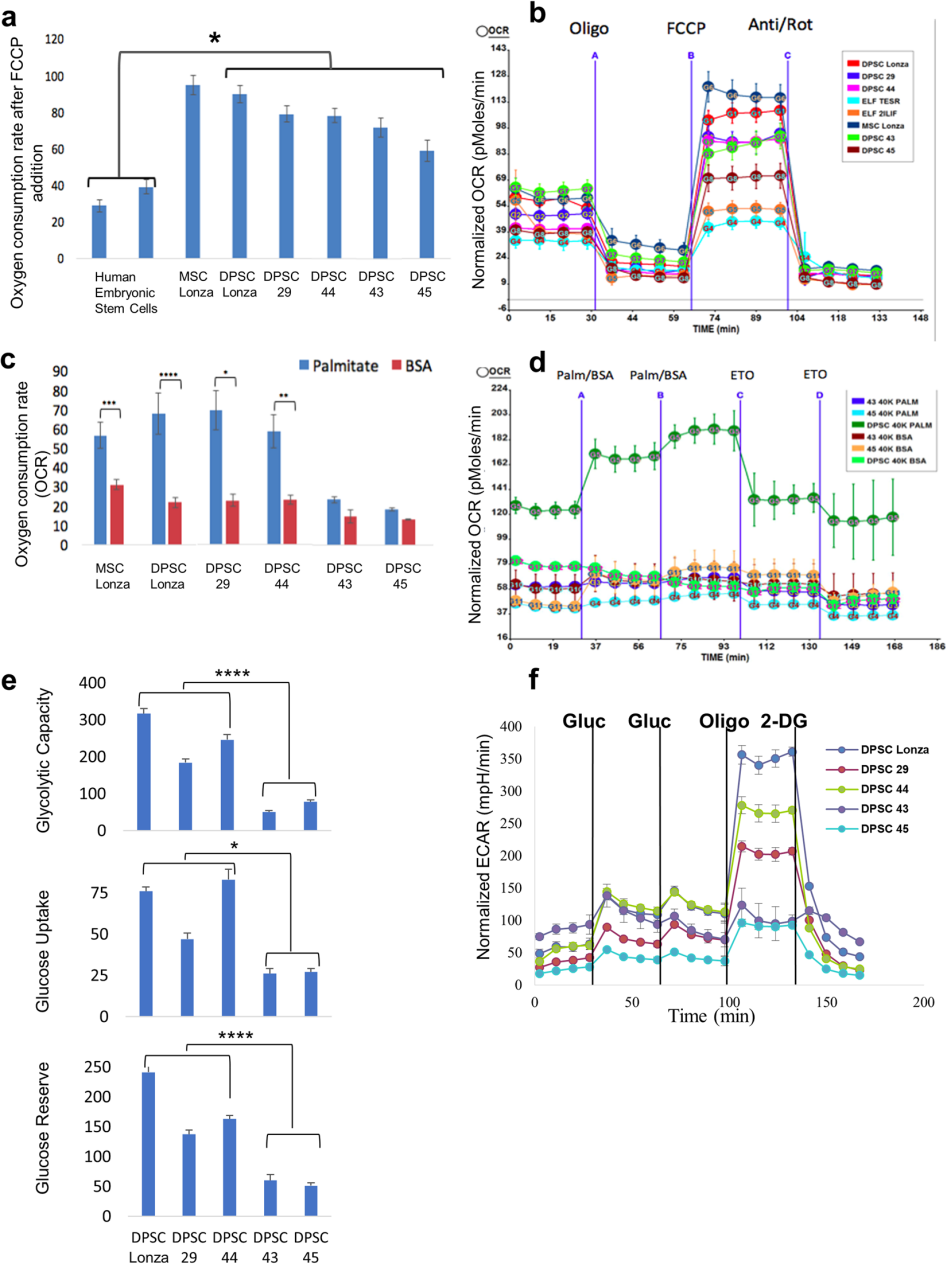
Metabolic assays showing the metabolic differences between the candidate DPSCs lines. (**a**,**b**) Mitostress Assay shows that DPSCs have higher mitochondrial activity than human embryonic stem cells (*p = 0.002). (**c**,**d**) From the selected candidate cell lines, DPSC 29 and 44 use palmitate inferring their ability to use fatty acid as an energy source. DPSC 43 and 45 have lower ability to use fatty acid as an energy source. (**e**,**f**) Glycolysis stress assay conducted on the selected candidates shows that DPSC 43 and 45 have significantly lower glycolytic capacity compared to DPSC 29, 44 and commercial DPSCs. Significance was determined by unpaired Student’s t-test; n = 3–12 per cell line; *p < 0.05; **p < 0.01; ***p < 0.001; ****p < 0.0001; Graph error bars are the means ± SEM.

Recent work has revealed that cellular metabolism plays other vital roles beyond simply the production of energy. In some situations, metabolism is shown to regulate cellular fate^72^. While pluripotent stem cells can switch their metabolic requirements to facilitate cellular changes and hematopoietic stem cells are regulated by metabolic changes^72,73^, very little is known about adult DPSCs metabolism, and if they utilize metabolism beyond cellular energy production. To analyse the metabolic signature of DPSCs, we used the Seahorse platform to study various metabolic aspects of these cells. The mitostress assay done by uncoupling the electron transport chain (ETC) and ATP synthase and treating the mitochondria with FCCP revealed that the maximum mitochondrial oxygen consumption rate (OCR) was higher in adult DPSCs than hESCs (Fig. 6a,b). The DPSC’s ability to use lipids as an energy source was measured by an increase in OCR with the presentation of palmitate as an energy substrate (Fig. 6c,d, and Supplementary Fig. S4). Their glycolytic capacity was assessed by measuring the extracellular acidification rate (ECAR) of the DPSCs after addition of glucose and thereafter treating the cells with a glucose analogue, 2Deoxy-D-glucose (2DG) to block glycolysis (Fig. 6f). The results showed that DPSCs were capable of utilizing both fatty acids and glucose as fuel for ATP production (Fig. 6c–f). However, interestingly, the RA DPSC lines 43 and 45 showed significant reduction in the utilization of fatty acids and glucose as energy sources compared to SA DPSC lines 29 and 44 (Fig. 6c–f). While the candidate cell lines included in this study showed comparable level of MSC markers CD146, and CD29, we wanted to address the potential correlation of these markers and the metabolic profile. We selected two more cell lines at random that showed higher expression than original candidate lines. We found that DPSC 125 that expresses CD29 at a higher level, and DPSC 120 that expresses both CD29 and CD146 at higher level showed similar capacity to utilize glucose as commercial DPSC Lonza (Supplementary Fig. S4).

### Rapid aging DPSC cell lines showed increase in TGF-β pathway activity and upregulation of cytoskeletal regulators

**Figure 7:**
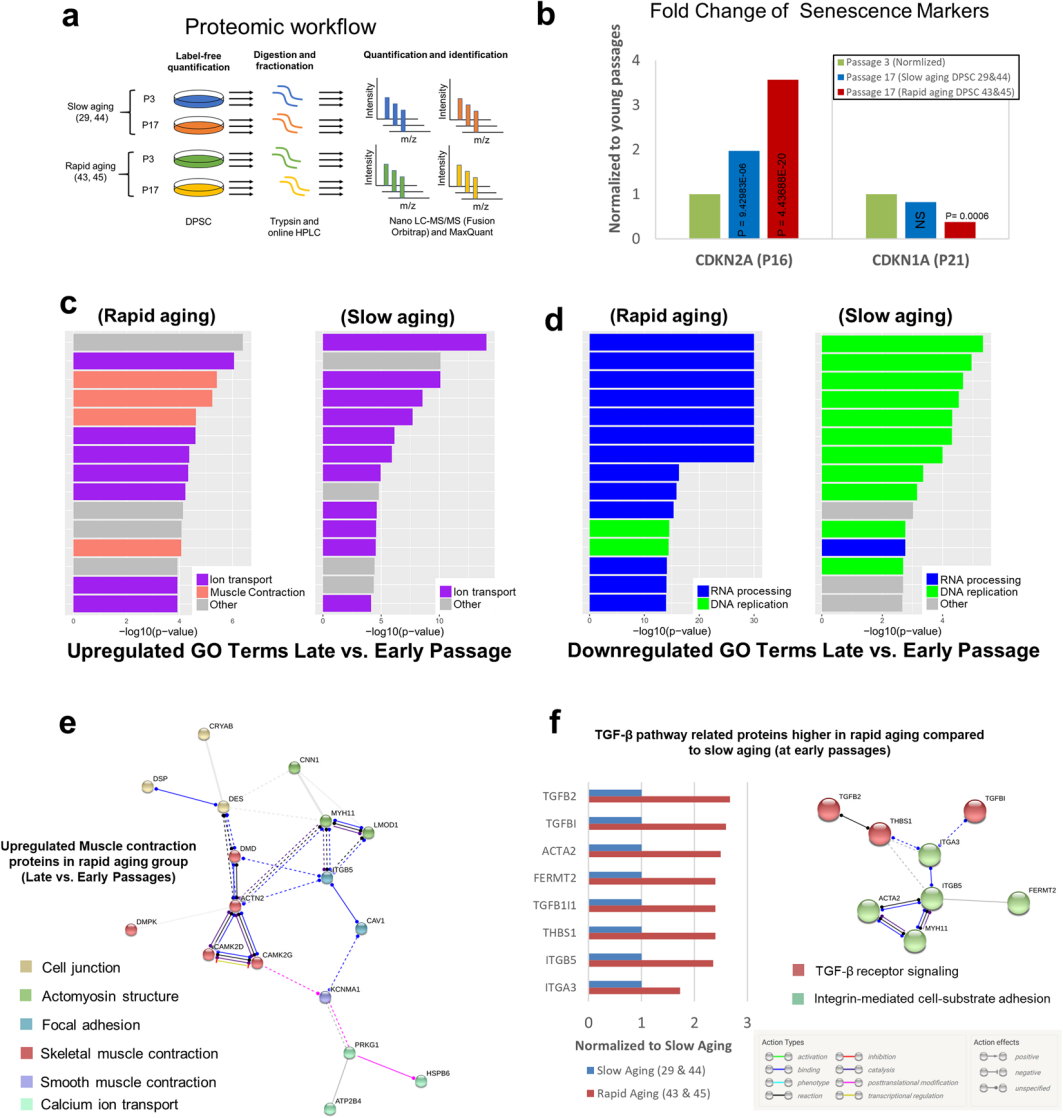
Proteomic analysis within the candidate cell lines. (**a**) Proteomic workflow used to identify differentially regulated protein expression in young (early passage) versus old (late passage) DPSC in rapid aging (lines 43, 45) or slow aging (lines 29, 44) cells. (**b**) Fold changes of replicative senescence markers, CDKN2A (known as P16) and CDKN1A (known as P21) in late passages of rapid aging cell lines (43 & 45) and slow aging cell lines (29 & 44). (**c**,**d**) Proteomics GO terms enrichment for slow aging (29 & 44) and rapid dividing (43 & 45) cell lines. (**e**) String analysis of enriched muscle contraction proteins expressed in late passages of rapid aging cell lines. (**f**) Fold change of TGF-β pathway related proteins higher in rapid aging compared to slow aging at early passages (P value < 0.05) (left), and String analysis of TGF-β pathway proteins expressed in early passages (P3) rapid aging cells at higher level than slow aging early passages (right).

To better understand the differences between the slow aging (SA: DPSC 29 and 44) and rapid aging cell lines (RA: DPSC 43 and 45) we analysed the proteomes of young/early passage (p3) and old/late passage (p17) cells of each class (Fig. 7a). We examined the data for two markers that had been previously suggested to mark levels of replicative senescence^57^, CDNK2A (Cyclin Dependent Kinase Inhibitor 2A, and also known as P16) and CDKN1A (Cyclin Dependent Kinase Inhibitor 1A, and also known as p21). CDNK2A is reported to increase with senescence. Our proteomic data confirmed an increase of CDKN2A in both groups but more significantly with the rapid aging group (p < 0.0001) (Fig. 7b). CDKN1A is reported to reach its maximum at the onset of growth arrest and subsequently decrease with senescence^57^, and we noted a similar trend of downregulation in both senesced groups, but significantly more in rapid aging cell lines (p = 0.0006) (Fig. 7b). These markers support the different kinetics for senescence observed for DPSC 43/45 and DPSC 29/44.

Unbiased GO-term analysis of the proteomic data showed significant enrichment of muscle contraction proteins, such as ACTN2 (Actinin Alpha 2), MYH11 (Myosin Heavy Chain 11, Smooth Muscle Isoform) and CNN1 (Calponin 1, Basic, Smooth Muscle) (Fig. 7c,d) in rapid aging cell lines (DPSC 43,45) when comparing the changes between late passage and early passage. Further analysis of the enriched muscle contraction proteins in String-analysis algorithm revealed the following functional clusters: cell junction regulators, actomyosin structure organization, focal adhesion, skeletal muscle contraction, smooth muscle contraction and calcium ion transport, all of which involved in controlling the cytoskeleton of the cells (Fig. 7e, Supplementary Dataset Sheet 5 and 6).

We hypothesized that the enrichment in muscle contraction proteins in rapid aging cells (RA-DPSC 43, 45) in late passages (p17), particularly the increase of the smooth muscle specific markers, CNN1 and MYH11, was an indication of differentiation towards myofibroblast-like cells^74,75^. It has been shown previously that fibroblasts^76,77^ or mesenchymal stem cells^78,79^ can differentiate into myofibroblast lineage when subjected to TGF-β pathway. Therefore, we examined our proteomics data further for TGF-β pathway related genes and found that some of the TGF-β pathway related proteins are enriched in rapid aging cells compared to slow aging cells in early passages (Fig. 7f, Supplementary Dataset Sheet 7). These proteins were clustered by String software into two functional categories: TGF-β receptor signalling, and Integrin-mediated cell-substrate adhesion. Of particular interest is ITGB5 (Integrin, Beta 5), a receptor for fibronectin, that has been found to mediate actin stress fiber formation^80^ by activating the latent form of TGF-β complex^81,82^. We also found that TGFB2 (an isoform of the TGF-β ligand) and the myofibroblast marker ACTA2^83^ (also known as α-smooth muscle actin, α-SMA) were expressed at a higher level in the rapid aging cell lines compared to slow aging cell lines in early passages (Fig. 7f) indicating a possible autocrine activation of TGF-β at early passages. These data further support our hypothesis that in rapid aging RA-DPSCs, TGF-β pathway and its muscle contraction gene targets were upregulated leading to spontaneous terminal differentiation towards myofibroblast-like cells in later passages.

### TGF-β pathway activation induces stress fiber formation and correlates with rapid DPSCs aging

**Figure 8:**
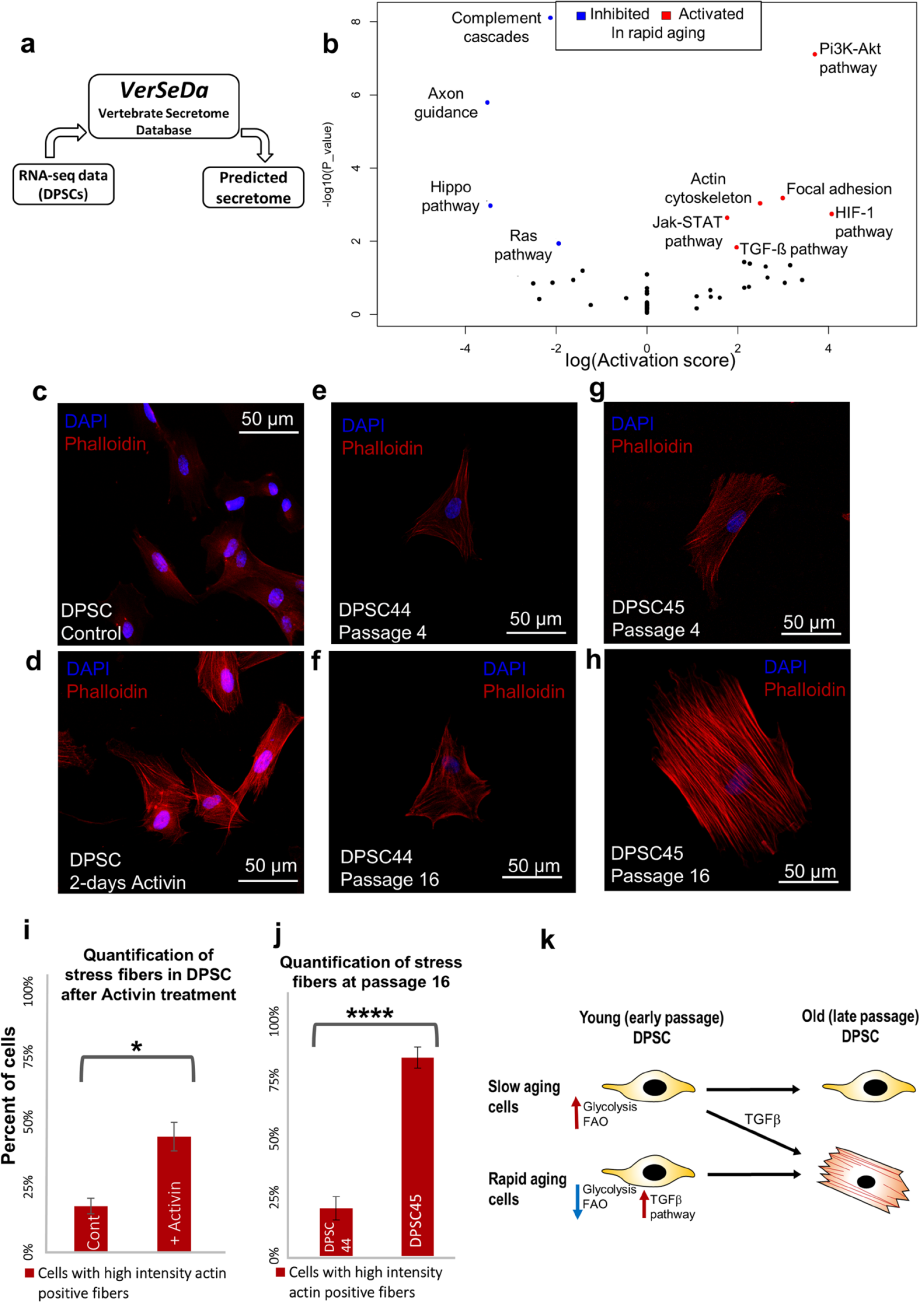
Activation of TGF-β pathway leads to formation of more actin stress fibers. (**a**) A simplified flowchart that shows the process of utilising VerSeDa secretome prediction utilities to analyse the predicted secretome in DPSC. (**b**) Signaling pathway impact analysis (SPIA) showing relatively activated or inhibited signaling pathways in rapid aging cell lines’ predicted secretome compared to slow aging cell lines. (False discovery rate < 0.1). (**c**,**d**) Activation of TGF-β pathway by adding Activin to the DPSCs growth media for 2-days increases actin stress fiber formation (**d**), compared to control (**c**). (**e**–**h**) Representative confocal images of DPSC 44 and DPSC 45 comparing the size and intensity of the actin fibers at passage 4, and passage16. (**i**) Quantification of stress fibers in early passage (P3) DPSC Lonza in control, and after treating with Activin for 2-days. Significance was determined by unpaired Student’s t-test; n = 100 cells were counted per condition; *p = 0.03; Graph error bars are the means ± SEM. (**j**) Quantification of cells with senescence phenotype at passage 16 in DPSC 44 and DPSC 45. Significance was determined by unpaired Student’s t-test; n = 100 cells were counted per condition; *p = 0.0005; Graph error bars are the means ± SEM. (**k**) Hypothetical model: DPSCs transcriptome and metabolic analysis suggest that low glycolysis and fatty acid oxidation (FAO) and upregulation of TGF-β activity at early passages are predictive for rapid aging phenotype in later passages.

To examine the possibility of an autocrine effect underlining the rapid aging phenotype, we analysed the predicted secretome of the young/early passage DPSCs, since secreted molecules can have an autocrine effect in long-term cultures^84^. We used VerSeDa online tool^55^ to predict the secretome of young DPSCs based on the transcriptomic data. We first used all expressed genes across different DPSC lines (Fig. 8a). The result contained a set of four thousand genes of secreted or transmembrane proteins. We performed gene ontology (GO) enrichment analysis to have an overview of the biological processes involved in DPSCs secretome (Supplementary Fig. S5). Some of the top GO terms were “extracellular matrix organization”, “cell adhesion”, and “positive regulation of cell migration” marking proteins which are expected to be in any MSCs secretome^85^. More interestingly, the GO term “response to hypoxia” including genes such as VEGFA (vascular endothelial growth factor A), and VEGFB, might explain the DPSCs sensitivity to hypoxia, as previously reported^86^. The GO term “semaphorin-plexin signalling pathway” which includes different families of semaphorin ligands & plexin receptors^87^ suggests that this pathway maybe used in DPSCs migration process towards the injury site^11^. DPSCs are also expressing NGF (Nerve Growth Factor) as previously reported^88^.

We further performed GO term enrichment analysis between secretome genes up-regulated in SA and RA cell lines at early passages. Terms such as “axon guidance” and “cell migration” were upregulated in slow aging cells, which include genes for multiple semaphorin family proteins, as well as BMP4 (bone morphogenetic protein 4) (Supplementary Fig. S5). Semaphorin 3E (SEMA3E), Semaphorin 4D (SEMA4D) and Semaphorin 5 A (SEMA5A) have been reported to inhibit focal adhesion by promoting the disassembly of integrin and actin stress fibers on multiple different cell types including glia, platelets, different types of immune cells, and a number of cancer cell lines, as well as promoting proliferation and cell migration of endothelial cells^89–92^. BMP4 has been suggested to attenuate the effect of TGF-β2 on the accumulation of extracellular matrix adhesion proteins and therefore aid in cell migration^93,94^. In contrast, terms such as “cell adhesion” and “chondrocyte development” were upregulated in rapid aging cells which include aggrecan(ACAN), fibronectin, integrin & osteonectin (SPARC), all of which are reported to be up-regulated under TGF-β pathway activation^78,95–102^ (Supplementary Fig. S5).

To further analyse the differences in the secretome between rapid aging cells and slow aging cells, we used Signalling Pathway Impact Analysis (SPIA) method^48^, since signalling pathways in particular are closely related to secretome of MSCs^103^. SPIA analyses the differentially expressed genes and their log fold changes while taking into consideration the signalling pathway network topology from KEGG database (Kyoto Encyclopedia of Genes and Genomes), in order to identify pathways relatively activated or inhibited in a given set of genes. The results from SPIA confirm that TGF-β pathway, focal adhesion and regulation of actin cytoskeleton are activated at the transcriptomic level in rapid aging cells compared to slow aging (Fig. 8b, Supplementary Dataset Sheet 8), which is what we found at the proteomic level as well.

To test the function of TGF-β pathway for activating actin stress fibers in DPSCs, we treated the SA DPSCs with Activin, and found a significant increase in actin stress fibers in SA DPSCs (Fig. 8c,d,i). Similar stress fibers were previously observed in fibroblasts (Fig. 5a). These data show that TGF-β pathway activation is sufficient for actin stress fiber formation in SA DPSCs and suggest that TGF-β pathway activation in RA DPSC results in stress fiber formation and premature senescence. We tested this by quantifying the cells with stress fibers in RA late passage DPSCs. Importantly, we observed a significant increase in stress fiber formation in RA cells, later passages (P value = 0.0001) (Fig. 8e–h,j–k). One of the inhibited pathways in RA cells is the Hippo pathway (Fig.  8b), which has been suggested to control cell proliferation of DPSCs^104^. Therefore, low Hippo pathway activity and TGF-β induced stress fiber formation may explain the slow division rate in RA cells compared to SA cells.

To further dissect the genes that contributed to the slow cell division, a transcriptome level analysis was performed comparing early passage slow vs. rapid aging cells. The genes which might be responsible for their aging and metabolic profile were identified (Supplementary Fig. S6, and Supplementary Table S8, Supplementary Dataset Sheet 4). Some of the key genes enriched in early passages of rapidly aging (RA) cell lines were cell cycle regulators. One of these genes, G0S2 is a G0/G1 switch protein also found to be associated with replicative senescence of human dermal fibroblasts^105^ and is known to maintain quiescence in hematopoietic cells^106^. Other enriched genes, GATA2 (GATA Binding Protein 2) and DDIT4 (DNA Damage Inducible Transcript 4) are also known to regulate quiescence in hematopoietic stem and progenitor cells (GATA2)^107^, or regulates mesenchymal stem cell fate through mTOR pathway (DDIT4)^108^. Interestingly G0S2 blocks lipolysis through direct interaction and inhibition of triglyceride hydrolase activity of Adipose triglyceride lipase^109^, and together with GATA2 are both regulated by PPAR-γ, connecting them to metabolic regulation. In the rapidly aging cell lines (RA-DPSC 43 and 45) the utilization of glucose or fatty acids as fuels for ATP production is significantly reduced compared to the SA cell lines, DPSC lines 29 and 44 (Fig. 6c–f). Importantly, this dramatic reduction in glycolytic capacity or fatty acid beta-oxidation was observed in DPSC 45 and 43 already at the early passages (P3). Therefore, the genes connected to metabolic regulation may be responsible for challenged lipid metabolism in rapidly aging cells and might promote terminal differentiation and thereby favour cellular senescence. These findings highlight the predictive metabolic signature for DPSCs aging *in vitro*. It will be important to investigate if metabolic signature is also predictive for DPSC aging *in vivo*. The identified predictive signals *in vitro* could aid in the selection of an optimal population of DPSCs to be used for *in vitro* expansion and further development of cell based therapy in the future.

## Discussion

Here we studied the aging of dental pulp stem cells (DPSCs), a population of adult stem cells that is known to participate in the repair of an injured tooth. Using high throughput transcriptomic and proteomic analysis we identified markers for DPSC populations with accelerated replicative senescence. In particular, we show that the transforming growth factor-beta (TGF-β) pathway and the cytoskeletal proteins are upregulated in rapid aging DPSCs, indicating a loss of stem cell characteristics and spontaneous initiation of terminal differentiation. Using metabolic flux analysis, we identified a metabolic signature for the rapid aging DPSCs, prior to onset of the senescence phenotypes. This metabolic signature is therefore predictive for rapid DPSCs aging.

It has been shown that mesenchymal stem cells age during *in vitro* expansion and exhibit characteristic hallmarks of aging such as replicative senescence and decrease in telomerase length^110^. We studied the effects of *in vitro* aging in DPSCs and observed that DPSCs showed differences in the rate of replicative senescence. A proteomic analysis of rapid and slow aging cells revealed that genes responsible for muscle contraction were upregulated in rapid aging DPSC (late passage cells vs early passage cells). We hypothesized that the rapid aging cells were differentiating into myofibroblasts-like cells in later passages. It has been shown previously that fibroblasts^76,77^ or mesenchymal stem cells^78^ can differentiate into myofibroblast lineage when subjected to TGF-β pathway activity. We confirmed the upregulation of TGF-β pathway related proteins in rapid aging cells compared to slow aging cells at early passages and validated that activation of TGF-β pathway significantly increased actin stress fibers in DPSCs. TGF-β1 induces mobilization and rapid polymerization of actin cytoskeleton and leads to the formation of stress fibers, a known morphological hallmark of cellular differentiation and aging^111–114^. More recently, treatment of DPSC with Notch ligand Jagged1 has shown to lead to reduced proliferation, upregulation of TGF-β and differentiation markers^115^, which could be correlated to our findings in rapid aging DPSCs. We now propose that upregulated TGF-β pathway genes may be considered as a hallmark of early onset of spontaneous differentiation in primary DPSCs in long-term cultures (Fig. 8).

Cellular metabolism has been implicated in cell fate determination and stem cell activity in a variety of different contexts^116–119^. Highly proliferative stem cells have unique metabolic requirements^120^, and they have the ability to switch between different metabolic pathways depending on changes in substrate availability^72^. Switching between different metabolic pathways can also regulate quiescent stem cell populations and the onset of differentiation^121–126^. We now show that adult stem cells, DPSCs, are metabolically highly active cells that can utilize multiple fuel sources for ATP production. Mitochondrial theory of aging posits that mitochondrial aging is a fundamental cause of cellular aging^127^. Accordingly, we find that the metabolic profile of DPSCs with early onset of cellular aging differ from the DPSCs with slow aging. In particular, DPSCs showing early onset of cellular aging exhibited lower glycolytic capacity and had highly reduced capacity to utilize lipids as an energy source. Importantly, these defects in preferred fuel usage were observed prior to the onset of other phenotypes, such as slow division rates. We therefore argue that metabolism serves as an early, predictive indicator of DPSCs tendency to lose stem cell self-renewal capacity. This raises the possibility that regulation of replicative senescence is controlled by similar switch in metabolism as seen previously with regulation of quiescence.

## Supplementary information

Supplementary Figures and Tables

Supplementary Dataset
