# Supplementary material for: Metabolism as an early predictor of DPSCs aging
Source: Sci Rep. 2019 Feb 18;9:2195. doi: 10.1038/s41598-018-37489-4 (PMC6379364; doi:10.1038/s41598-018-37489-4)
Supplement: Supplementary file 1 — Supplementary Figures and Tables [file 41598_2018_37489_MOESM1_ESM.pdf]

Metabolism as an early predictor of DPSCs aging

Dannie Macrin[1,2,3,+], Ammar Alghadeer[2,4,5,+], Yan Ting Zhao[1,2,4], Jason W. Miklas[2,6], Abdiasis M. Hussein[1,2], Damien Detraux[1,2], Aaron M. Robitaille[2,7], Anup Madan[8], Randall T. Moon[2,7], Yuliang Wang[2,9], Arikketh Devi[2,3], Julie Mathieu[1,2,10], and Hannele Ruohola-Baker [1,2,4,6,*]

Supplementary Figure S1

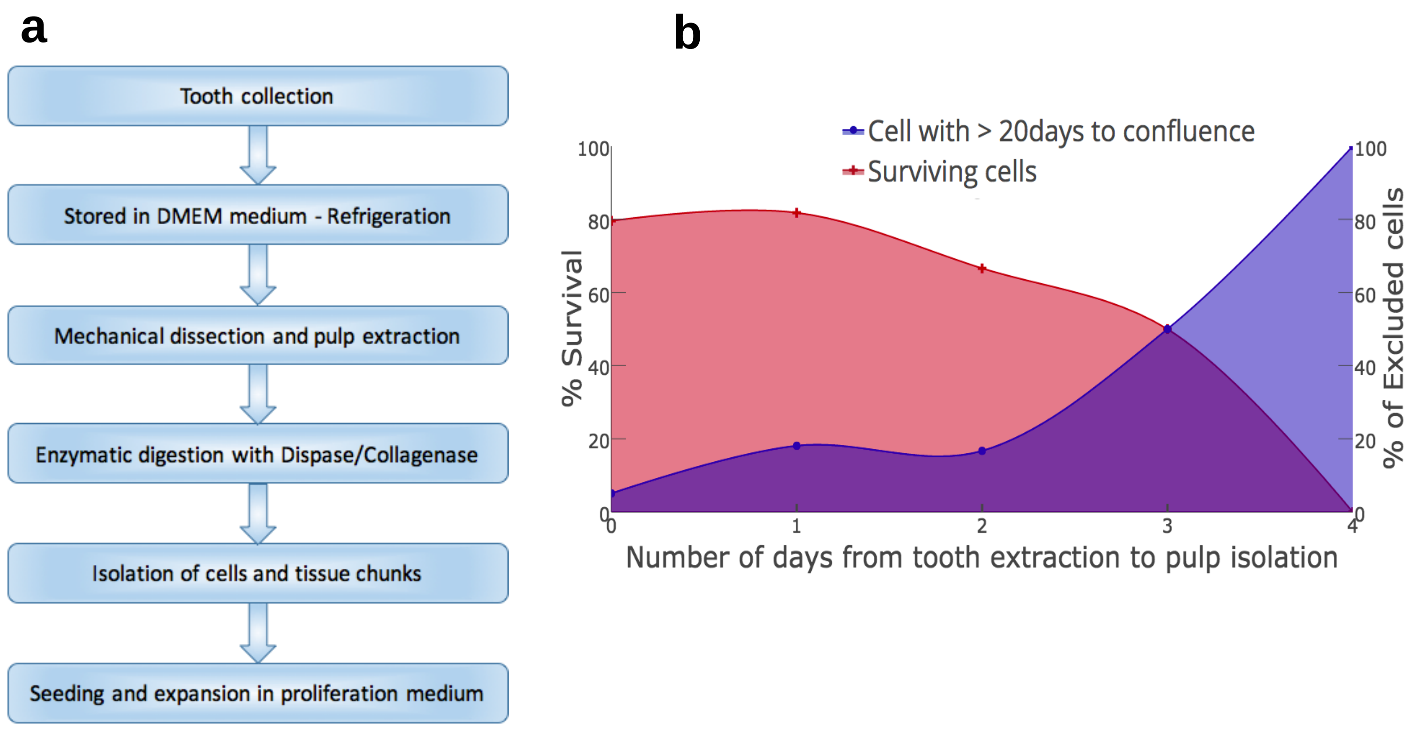

Supplementary Figure S1: (a) A simplified flowchart of the extraction protocol. (b) The survivability of the cells decreased with increase in number of days between tooth extraction and pulp isolation which was evident loss/decrease in proliferative capacity.

Supplementary Figure S2

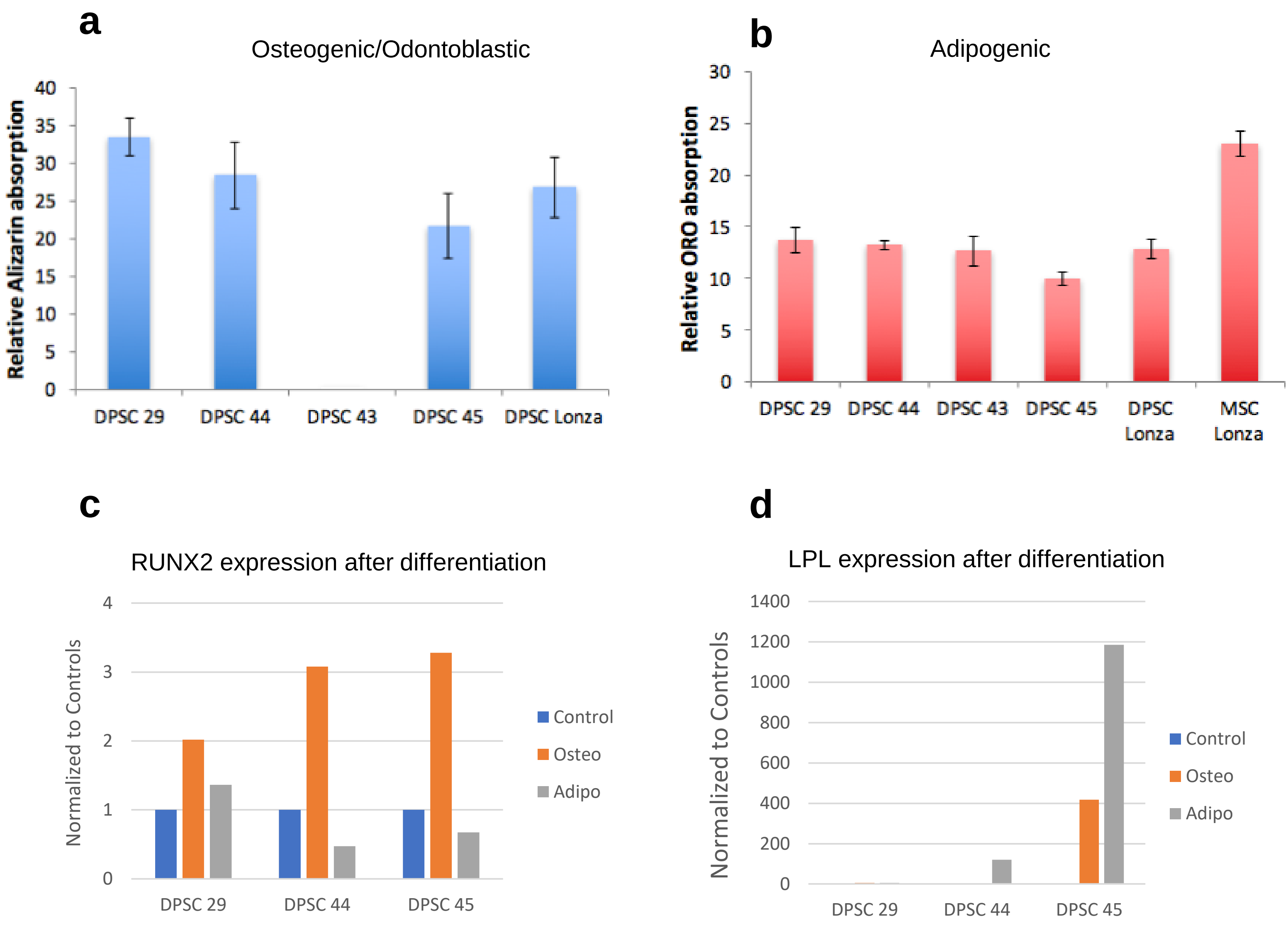

Supplementary Figure S2: Biological repeats and molecular markers of differentiation. (a) Biological repeats of Alizarin staining of osteo/odontogenesis. (b) Biological repeats of Oil Red O staining of Adipogenesis. Graph error bars are the means ± SEM. (c & d) Quantitative PCR reveals the expression of differentiation markers: (c) Runx2, which marks early osteo/odontogenesis, (d) Lipoprotein lipase (LPL), which mark adipogenesis.

Supplementary Figure S3

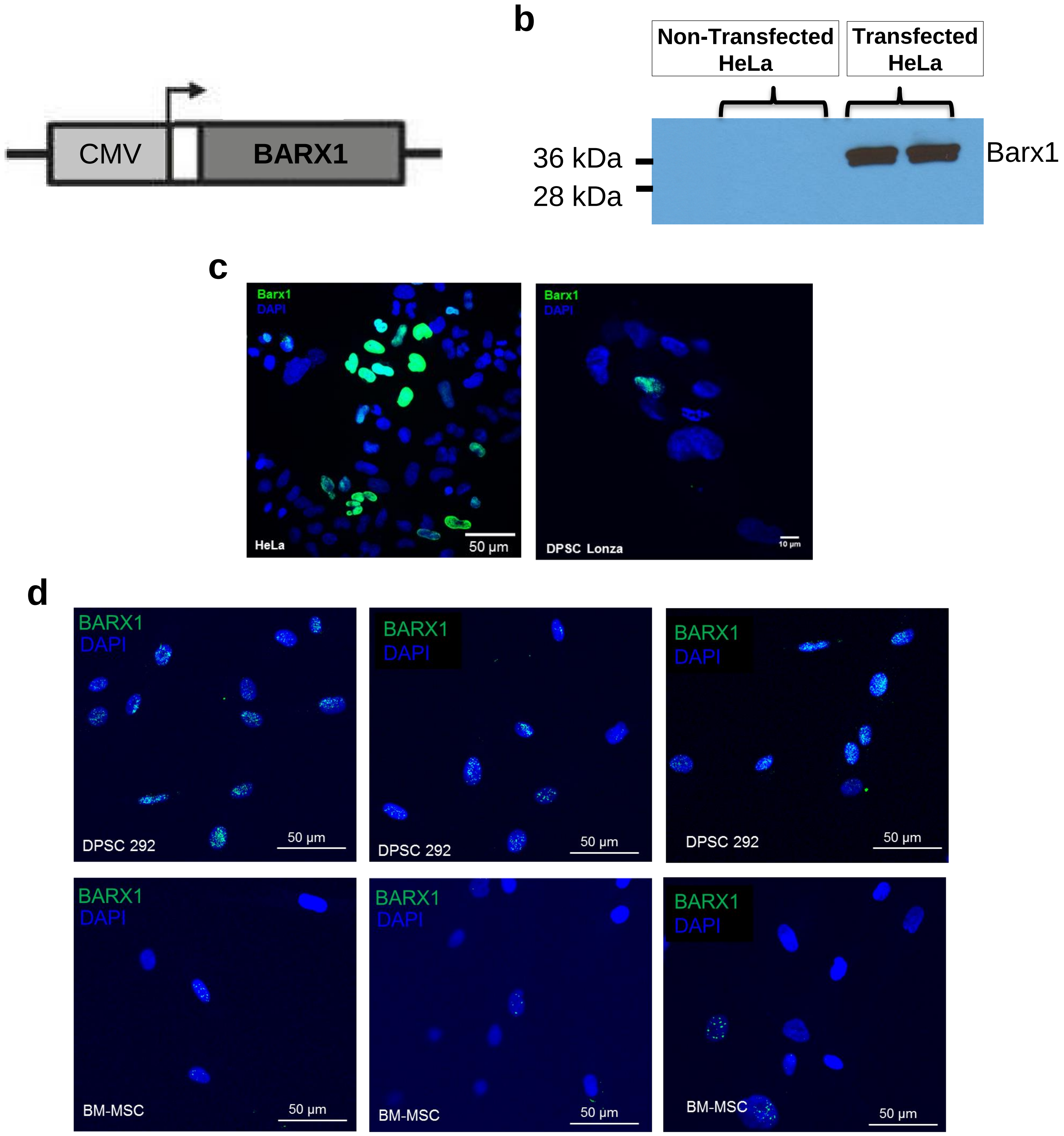

Supplementary Figure S3: Validation of Barx1 antibody by overexpression plasmid transfected into HeLa cells. (a) The overexpression construct of BARX1 gene under the control of CMV promotor in pcDNA 3.1+ plasmid (supplied by Genescript). (b) The overexpression of Barx1 in transfected HeLa cells was validated with Western Blot. (c) Immunofluorescent staining of Barx1 in HeLa cells, and DPSC Lonza showing the nuclear localization of Barx1 in the transfected cells. Exposure settings and laser intensity of the confocal microscope were adjusted for the overexpression signal in HeLa cells, and same settings were used for Lonza DPSCs. (d) Immunofluorescent staining of Barx1 in DPSC 292 and BM-MSCs Lonza. Exposure settings and laser intensity of the confocal microscope were adjusted for DPSC 292.

Supplementary Figure S4

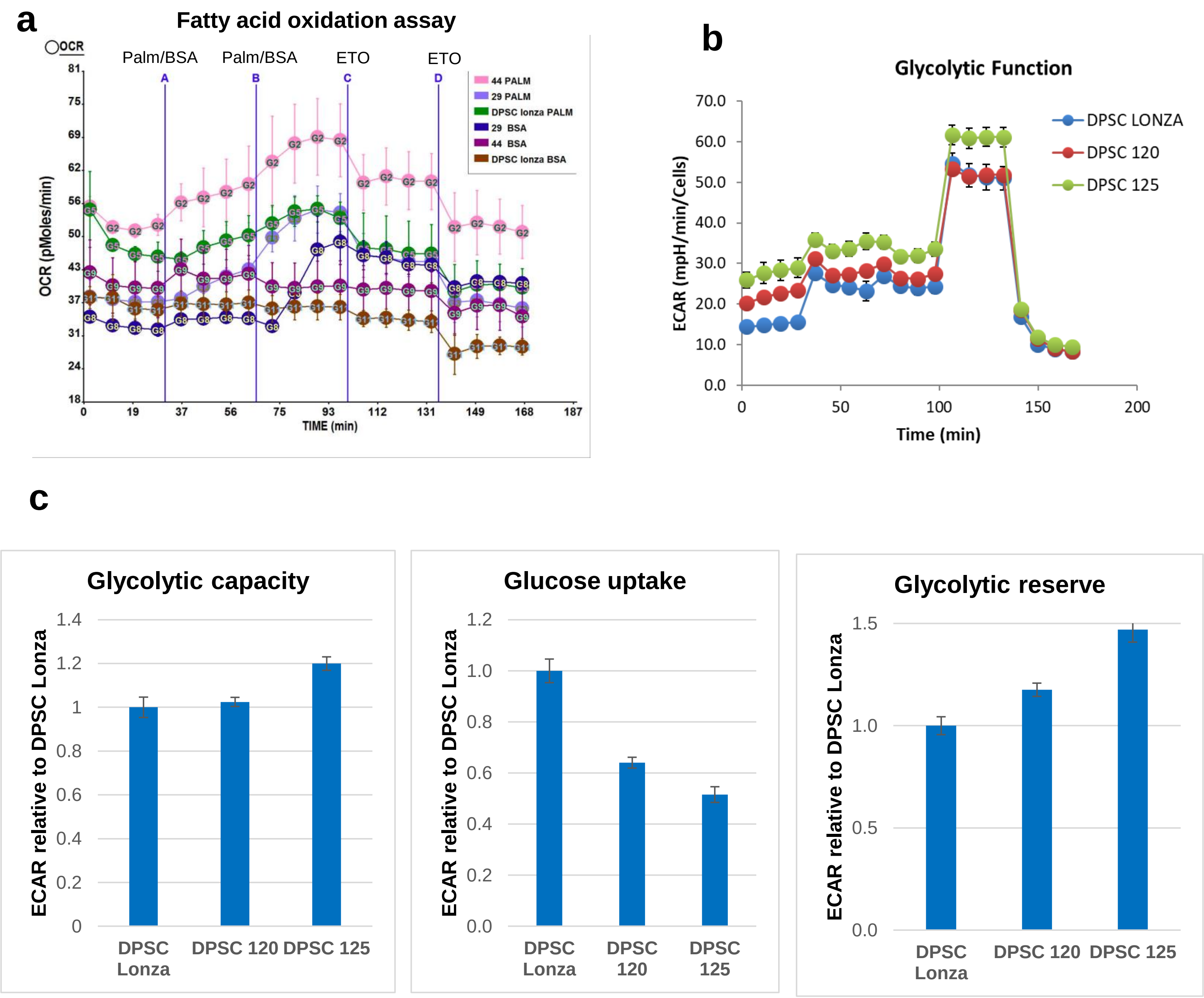

Supplementary Figure S4: (a) Representative graphs for palmitate assay of DPSC 29 and DPSC 44 inferring that they were able to use lipids as a source of energy. Graph error bars are the means ± SEM. (b, c) Representative graphs for glucose stress assay of DPSC 120 and DPSC 125 inferring that they were able to use glucose as a source of energy normally compared to DPSC Lonza. Graph error bars are the means ± SEM.

Supplementary Figure S5

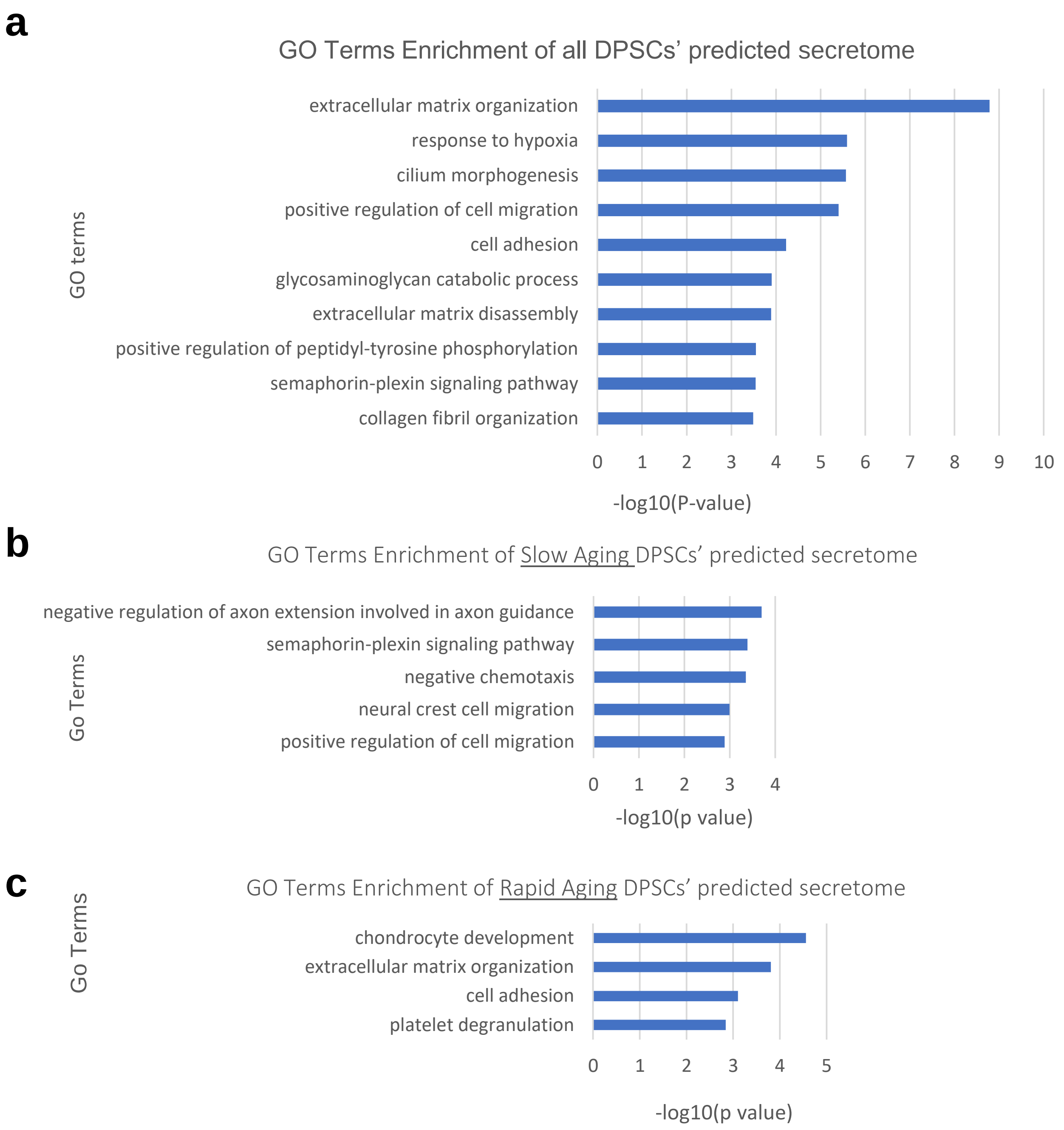

Supplementary Figure S5: Gene ontology (GO) enrichment for predicted secretome. (a) GO terms of biological processes enriched across different DPSC lines' predicted secretome. (b) GO terms of biological processes based on significantly upregulated genes in slow aging DPSCs predicted secretome compared to rapid aging lines. (c) GO terms of biological processes based on significantly upregulated genes in rapid aging DPSCs' predicted secretome compared to slow aging lines.

Supplementary Figure S6

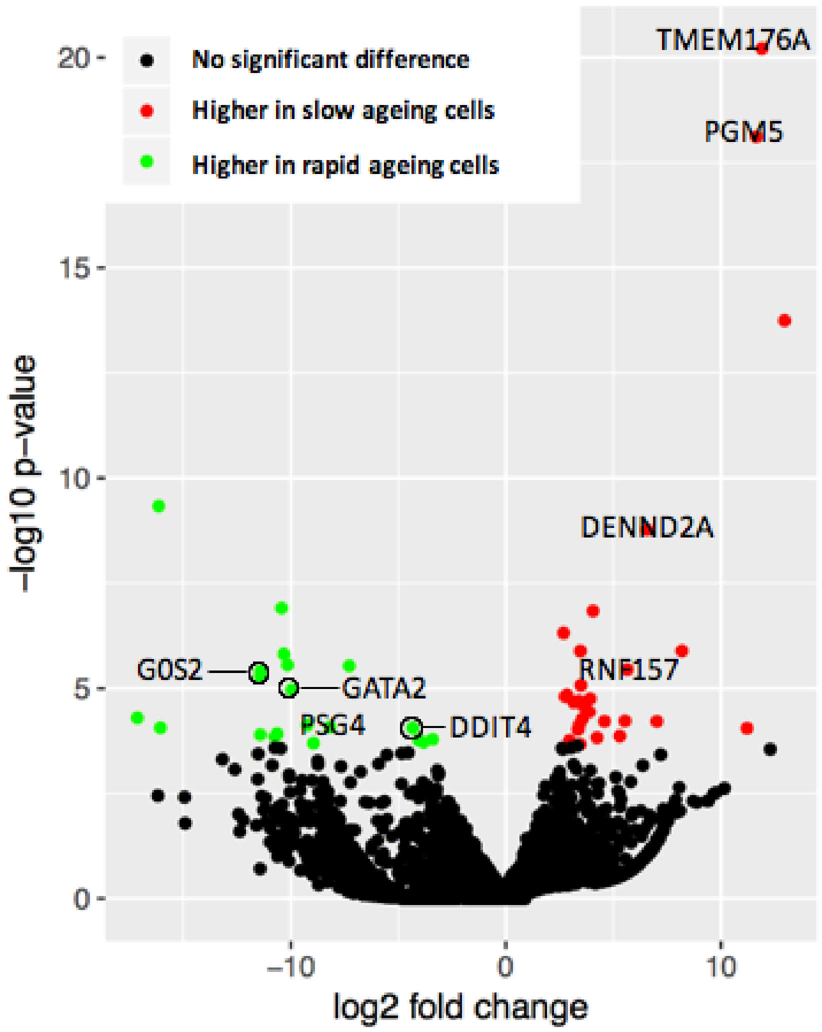

Supplementary Figure S6: Volcano plot of genes differentially expressed in cells with slow aging phenotype [DPSC 29 (P4) & DPSC 44 (P5)] compared to rapid aging phenotype [DPSC 43 & 45 (P4)].

Metabolism as an early predictor of DPSCs aging

Dannie Macrin[1,2,3,+], Ammar Alghadeer[2,4,5,+], Yan Ting Zhao[1,2,4], Jason W. Miklas[2,6], Abdiasis M. Hussein[1,2], Damien Detraux[1,2], Aaron M. Robitaille[2,7], Anup Madan[8], Randall T. Moon[2,7], Yuliang Wang[2,9], Arikketh Devi[2,3], Julie Mathieu[1,2,10], and Hannele Ruohola-Baker [1,2,4,6,*]

Supplementary Table S1

Serum free media (SFM)			
Components	Supplier information	Stock Concentration	Final concentration
MCDB-201 media	Sigma M-6770	1L sachet powder	40% v/v
LA-BSA	Sigma L-9530	100x	1x
ITS	Sigma I-3146	100x	1x
PenStep	Hyclone SH30010	100x	1x
2P L-Ascorbic Acid	Sigma A-8960	10mM	0.1mM
DMEM-LG	Gibco 11885092	-	62% v/v
Dexamethasone	Sigma D-2915	250μM	50nM

Supplementary Table S2

MAPC Osteoblast Differentiation Media			
Components	Supplier information	Stock Concentration	Final concentration
Serum Free Media			78% v/v
FBS			10%
β- Glycerophosphate	CalBiochem 36575	0.1M	10mM
2P L-Absorbic Acid	A-8960	0.1M	0.2mM
Dexamethasone	Sigma D-2915	250μM (H_2O)	100nM

Supplementary Table S3

MAPC Adipocyte Differentiation Media			
Components	Supplier information	Stock Concentration	Final concentration
Serum Free Media			88.5% v/v
Horse Serum			10%
Indomethacin	Alfa Aesar A19910	10mM	100μM
Dexamethasone	Sigma D-2915	250μM (H_2O)	1μM
3-isobutyl-1-methyl-xanthine (IBMX)	ACROS 228420010	0.5M	0.5mM

Supplementary Table S4

qPCR Primers		
Genes	Primers	Sequences (5'-3')
Beta-Actin (NM_001101)	Forward	TCCCTGGAGAAGAGCTACG
	Reverse	GTAGTTTCGTGGATGCCACA
RUNX2 (NM_001024630.3)	Forward	TGGAACATCTCCATCAAGGCAG
	Reverse	TCAGGATATTCGGGACGTTGGA
LPL (NM_000237)	Forward	ATGGAGAGCAAAGCCCTGCTC
	Reverse	GTTAGGTCCAGCTGGATCGAG
BARX1 (NM_021570)	TaqMan	Applied Biosystems [Hs00222053_m1] Catalog Number: 4331182

Supplementary Table S5

Differentially expressed genes in ESC vs MSC/DPSC

HIGHLY EXPRESSED IN	Gene Symbol	Gene Name	Description
Embryonic Stem cells (ESCs)	TDGF1	Teratocarcinoma Derived Growth Factor 1	Embryonic marker [128]
	SOX2	Sex Determining Region Y-Box 2	Embryonic marker [128]
	NANOG	Homeobox Transcription Factor Nanog	Embryonic marker [128]
	POUF51	POU Class 5 Homeobox 1	Embryonic marker [128]
	LIN28A	Zinc Finger CCHC Domain-Containing Protein 1	Let 7 associated protein responsible for growth and metabolism in ESCs [129]
	TRIM71	Tripartite Motif Containing 71	RNA binding protein important for pluripotency [130]
	DPPA4	Developmental Pluripotency Associated 4	Involved in the maintenance of pluripotency in stem cells [131]
Mesenchymal Stem cells (MSC/DPSC)	CD248	Endosialin	Mesenchymal marker [132]
	CD73	ecto-5'-nucleotidase	Mesenchymal marker [133]
	CD29 (ITGB1)	Integrin beta 1	Mesenchymal marker [133]
	KRTAP 1-5	Keratin Associated Protein 1-5	expression increase after actin depolarization and can alter differentiation capacities of MSC [134]
	FAM180A	Family with Sequence Similarity 180 Member A	Observed in MSCs and *in vitro* progenies [135]
	SPOCD1	SPOC Domain Containing 1	Gene involved in cell proliferation [136]

Supplementary Table S6

Genes over-expressed in MSC/DPSC compared to Fibroblasts

HIGHLY EXPRESSED IN	Gene Symbol	Gene Name	Description
MSC/DPSC	BARX1	BarH-Like Homeobox 1	Role in patterning of teeth [6,59,60]
	LIF	Leukemia Inhibitory Factor	member of the IL-6 cytokine family that activate STAT3[137,138]
	ALPL	Alkaline Phosphatase	Bone mineralization [139]
	TBX2	T- Box 2	DNA binding protein responsible for epithelial to mesenchymal transition [140]
	JUND	Transcription Factor Jun-D	Protects against P53 dependent senescence and apoptosis [141]
	GDF5	Growth Differentiation Factor 5	Promotes osteogenic potential of bone marrow mesenchymal stem cells [142]
	MSX1	Msh Homeobox 1	Protein associated with osteogenesis and odontogenesis of DPSCs [139]
	CMKLR1	Chemerin Chemokine-Like Receptor 1	Involved in osteogenesis and adipogenesis of bone marrow stem cells [143]

Supplementary Table S7

Differentially expressed genes in MSC vs DPSC

HIGHLY EXPRESSED IN	Gene Symbol	Gene Name	Description
MSCs	COMP	Cartilage Oligomeric Matrix Protein	Enhances matrix assembly during chondrogenesis [144]
	GATA6	GATA Binding Protein 6	Involved in differentiation of ESCs [145]
	ADAMTS15	ADAM Metallopeptidase with Thrombospondin Type 1 Motif 15	Skeletal Muscle development
	SCRG1	Stimulator Of Chondrogenesis 1	Involved in self-renewal, migration, and osteogenic differentiation potential in mesenchymal stem cells [146]
	BST2	Bone Marrow Stromal Cell Antigen 2	Bone marrow stem cells marker enriched in MSCs [146]
DPSCs	CHN1	Chimerin 1	Predominantly expressed in neurons
	MXRA5	Matrix Remodeling Associated 5	Predominantly expressed in multiple cancer cells
	FBN2	Fibrillin 2	Regulates osteoblast supported osteoclasts [147]
	MSX1	Homeobox Protein Hox-7	Protein responsible for osteogenic and odontogenic differentiation of DPSCs [139]
	SFRP1	Secreted Frizzled Related Protein 1	Protein responsible for maintenance of hematopoietic mesenchymal stem cells [148]
	BMP2	Bone Morphogenetic Protein 2	Plays central role in osteoblast differentiation
	CD274	B7 Homolog 1	Novel mesenchymal marker [132]

Supplementary Table S8

Differentially expressed genes in Rapid ageing vs Slow ageing phenotypes

HIGHLY EXPRESSED IN	Gene Symbol	Gene Name	Description
Rapidly ageing cells	G0S2	G0/G1 Switch 2	Found to be associated with replicative senescence of human dermal fibroblasts [149]
	GATA2	GATA Binding Protein 2	known to regulate quiescence in hematopoietic stem and progenitor cells [150] [151]
	PSG4	Pregnancy Specific Beta-1-Glycoprotein 4	
	DDIT4	DNA Damage Inducible Transcript 4	DNA damage inducible protein which regulates mesenchymal stem cell fate [152]
	CMKLR1	Chemerin Chemokine-Like Receptor 1	Involved in osteogenesis and adipogenesis of bone marrow stem cells [143]
Slow ageing cells	TMEM176 A&B	Transmembrane Protein 176 A&B	Present in neoplastic fibroblasts and cancer cells [153]
	PGM5	Phosphoglutamase 5	Lack enzymatic activity [154], but might have structural roles[155]
	DENND2A	DENN Domain Containing 2A	MADD domain containing gene which is an apoptotic signal over expressed in neoplastic cells
	RFN157	Ring Finger protein 157	Maintain survival and morphology of cultured neuronal cells [156]

Supplementary References

128 Fang, Z. F. *et al.* Human embryonic stem cell lines derived from the Chinese population. *Cell research* **15**, 394-400, doi:10.1038/sj.cr.7290307 (2005).

129 Shyh-Chang, N. & Daley, G. Q. Lin28: primal regulator of growth and metabolism in stem cells. *Cell stem cell* **12**, 395-406, doi:10.1016/j.stem.2013.03.005 (2013).

130 Ye, J. & Blelloch, R. Regulation of pluripotency by RNA binding proteins. *Cell stem cell* **15**, 271-280, doi:10.1016/j.stem.2014.08.010 (2014).

131 Tung, P. Y., Varlakhanova, N. V. & Knoepfler, P. S. Identification of DPPA4 and DPPA2 as a novel family of pluripotency-related oncogenes. *Stem cells (Dayton, Ohio)* **31**, 2330-2342, doi:10.1002/stem.1526 (2013).

132 Camilleri, E. T. *et al.* Identification and validation of multiple cell surface markers of clinical-grade adipose-derived mesenchymal stromal cells as novel release criteria for good manufacturing practice-compliant production. *Stem cell research & therapy* **7**, 107, doi:10.1186/s13287-016-0370-8 (2016).

133 T, L. R. *et al.* MSC surface markers (CD44, CD73, and CD90) can identify human MSC-derived extracellular vesicles by conventional flow cytometry. *Cell communication and signaling : CCS* **14**, 2, doi:10.1186/s12964-015-0124-8 (2016).

134 Chang, T. H. *et al.* The effects of actin cytoskeleton perturbation on keratin intermediate filament formation in mesenchymal stem/stromal cells. *Biomaterials* **35**, 3934-3944, doi:10.1016/j.biomaterials.2014.01.028 (2014).

135 Ghazanfari, R. *et al.* Human Primary Bone Marrow Mesenchymal Stromal Cells and Their in vitro Progenies Display Distinct Transcriptional Profile Signatures. *Scientific reports* **7**, 10338, doi:10.1038/s41598-017-09449-x (2017).

136 Liang, J., Zhao, H., Hu, J., Liu, Y. & Li, Z. SPOCD1 promotes cell proliferation and inhibits cell apoptosis in human osteosarcoma. *Molecular medicine reports* **17**, 3218-3225, doi:10.3892/mmr.2017.8263 (2018).

137 Cherepkova, M. Y., Sineva, G. S. & Pospelov, V. A. Leukemia inhibitory factor (LIF) withdrawal activates mTOR signaling pathway in mouse embryonic stem cells through the MEK/ERK/TSC2 pathway. *Cell death & disease* **7**, e2050, doi:10.1038/cddis.2015.387 (2016).

138 Onishi, K. & Zandstra, P. W. LIF signaling in stem cells and development. *Development (Cambridge, England)* **142**, 2230-2236, doi:10.1242/dev.117598 (2015).

139 Goto, N. *et al.* Role of MSX1 in Osteogenic Differentiation of Human Dental Pulp Stem Cells. *Stem cells international* **2016**, 8035759, doi:10.1155/2016/8035759 (2016).

140 Wang, B. *et al.* The T box transcription factor TBX2 promotes epithelial-mesenchymal transition and invasion of normal and malignant breast epithelial cells. *PloS one* **7**, e41355, doi:10.1371/journal.pone.0041355 (2012).

141 Weitzman, J. B., Fiette, L., Matsuo, K. & Yaniv, M. JunD protects cells from p53-dependent senescence and apoptosis. *Molecular cell* **6**, 1109-1119 (2000).

142 Shimaoka, H. *et al.* Recombinant growth/differentiation factor-5 (GDF-5) stimulates osteogenic differentiation of marrow mesenchymal stem cells in porous hydroxyapatite ceramic. *Journal of biomedical materials research. Part A* **68**, 168-176, doi:10.1002/jbm.a.20059 (2004).

143 Muruganandan, S., Roman, A. A. & Sinal, C. J. Role of chemerin/CMKLR1 signaling in adipogenesis and osteoblastogenesis of bone marrow stem cells. *Journal of bone and mineral research : the official journal of the American Society for Bone and Mineral Research* **25**, 222-234, doi:10.1359/jbmr.091106 (2010).

144 Haleem-Smith, H., Calderon, R., Song, Y., Tuan, R. S. & Chen, F. H. Cartilage oligomeric matrix protein enhances matrix assembly during chondrogenesis of human mesenchymal stem cells. *Journal of cellular biochemistry* **113**, 1245-1252, doi:10.1002/jcb.23455 (2012).
145 Fujikura, J. *et al.* Differentiation of embryonic stem cells is induced by GATA factors. *Genes & development* **16**, 784-789, doi:10.1101/gad.968802 (2002).
146 Aomatsu, E. *et al.* Novel SCRG1/BST1 axis regulates self-renewal, migration, and osteogenic differentiation potential in mesenchymal stem cells. *Scientific reports* **4**, 3652, doi:10.1038/srep03652 (2014).
147 Nistala, H., Lee-Arteaga, S., Smaldone, S., Siciliano, G. & Ramirez, F. Extracellular microfibrils control osteoblast-supported osteoclastogenesis by restricting TGF{beta} stimulation of RANKL production. *The Journal of biological chemistry* **285**, 34126-34133, doi:10.1074/jbc.M110.125328 (2010).
148 Renstrom, J. *et al.* Secreted frizzled-related protein 1 extrinsically regulates cycling activity and maintenance of hematopoietic stem cells. *Cell stem cell* **5**, 157-167, doi:10.1016/j.stem.2009.05.020 (2009).
149 Yoon, I. K. *et al.* Exploration of replicative senescence-associated genes in human dermal fibroblasts by cDNA microarray technology. *Exp Gerontol* **39**, 1369-1378, doi:10.1016/j.exger.2004.07.002 (2004).
150 Tipping, A. J. *et al.* High GATA-2 expression inhibits human hematopoietic stem and progenitor cell function by effects on cell cycle. *Blood* **113**, 2661-2672, doi:10.1182/blood-2008-06-161117 (2009).
151 Endoh, M. *et al.* Coordinate expression of the human pregnancy-specific glycoprotein gene family during induced and replicative senescence. *Biogerontology* **10**, 213-221, doi:10.1007/s10522-008-9173-3 (2009).
152 Gharibi, B., Ghuman, M. & Hughes, F. J. DDIT4 regulates mesenchymal stem cell fate by mediating between HIF1alpha and mTOR signalling. *Scientific reports* **6**, 36889, doi:10.1038/srep36889 (2016).
153 Degnim, A. C. *et al.* Histologic findings in normal breast tissues: comparison to reduction mammaplasty and benign breast disease tissues. *Breast cancer research and treatment* **133**, 169-177, doi:10.1007/s10549-011-1746-1 (2012).
154 Moiseeva, E. P., Belkin, A. M., Spurr, N. K., Koteliansky, V. E. & Critchley, D. R. A Novel Dystrophin/Utrophin-Associated Protein is an Enzymatically Inactive Member of the Phosphoglucomutase Superfamily. *The FEBS journal* **235**, 103-113 (1996).
155 Molt, S. *et al.* Aciculin interacts with filamin C and Xin and is essential for myofibril assembly, remodeling and maintenance. *Journal of cell science* **127**, 3578-3592, doi:10.1242/jcs.152157 (2014).
156 Matz, A. *et al.* Regulation of neuronal survival and morphology by the E3 ubiquitin ligase RNF157. *Cell death and differentiation* **22**, 626-642, doi:10.1038/cdd.2014.163 (2015).
